# Randomized placebo-controlled clinical trial investigating the effect of antioxidants and a vasodilator on overall safety and residual hearing preservation in cochlear implant patients

**DOI:** 10.1186/s13063-020-04522-9

**Published:** 2020-07-14

**Authors:** Verena Scheper, Melanie Schmidtheisler, Florian Lasch, Heiko von der Leyen, Armin Koch, Jana Schwieger, Andreas Büchner, Anke Lesinski-Schiedat, Thomas Lenarz

**Affiliations:** 1 Department of Otolaryngology, Hannover Medical School, Carl-Neuberg-Str. 1, 30625 Hannover, Germany; 2 Cluster of Excellence Hearing4all, Oldenburg, Germany; 3 Institute for Biostatistics, Hannover Medical School, Carl-Neuberg-Str. 1, 30625 Hannover, Germany; 4 Hannover Clinical Trial Center, Carl-Neuberg-Str. 1, 30625 Hannover, Germany

**Keywords:** Free radical scavengers, Antioxidants, Cochlear implant, Hearing loss, Hearing pill, Hearing preservation, Residual hearing preservation, Vitamins

## Abstract

**Background:**

The standard therapy for patients suffering from sensorineural hearing loss is cochlear implantation. The insertion of the electrode array into the cochlea, with potential mechanical trauma and the presence of this foreign body inside the cochlea, may lead to free radical formation and reduced blood perfusion of the cochlea which can result in a loss of residual hearing. Studies have suggested that a particular combination of the antioxidants vitamins A, C and E as well as the vasodilator magnesium (together: ACEMg) may protect the residual hearing.

**Methods:**

The potential protective effect of ACEMg on residual hearing preservation in cochlear implant (CI) patients was investigated in a single-centre, randomized, placebo-controlled, double-blind phase II clinical trial. CI candidates with some residual hearing in low frequencies receiving MED-EL implants of different FLEX electrode array lengths were treated with ACEMg tablets or placebo respectively 2 days preoperatively and up to 3 months postoperatively. The study objective was to demonstrate that ACEMg is more efficacious than placebo in preserving residual hearing during cochlear implantation by comparing the hearing loss (change in hearing thresholds at 500 Hz from baseline) 3 months after the first fitting between the two treatment groups and to investigate the treatments’ safety.

**Results:**

Fifty-one patients were included in the study, which had to be terminated before the recruitment goal was reached because of IMP-resupply mismanagement of one partner. In the intention-to-treat population, 25 patients were treated with ACEMg and 24 patients with placebo. The mean hearing loss at 500 Hz was (± 15.84) 30.21 dB (placebo) or (± 17.56) 26.00 dB (ACEMg) 3 months after the initial fitting. Adjusting the postoperative hearing loss for the baseline residual hearing, planned electrode length and surgeon results in 8.01 dB reduced hearing loss in ACEMg-treated patients compared to placebo-treated ones. The safety analysis revealed that ACEMg was generally well-tolerated with adverse event frequencies below the placebo level.

**Conclusion:**

This is the first clinical trial investigating a drug effect on residual hearing in CI patients. These first-in-man data may suggest that a perioperative oral administration of ACEMg is safe and may provide protection of residual hearing in CI patients.

**Trial registration:**

EU Clinical Trial Register No. 2012-005002-22. Registered on 6 December 2013. Funding: European Commission FP7-HEALTH-2012-INNOVATION-2.

## Background

**Fig. 1:**
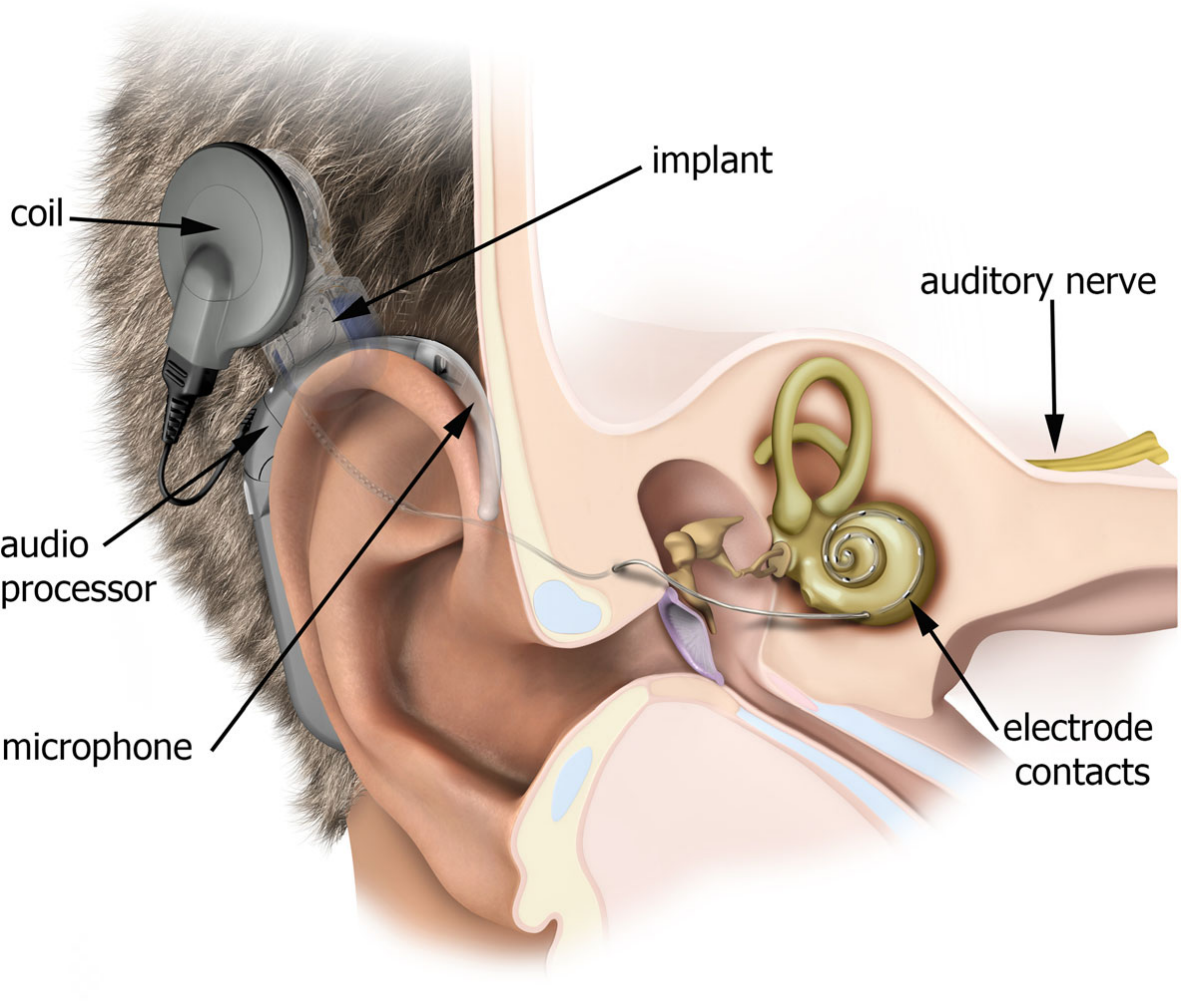
Cochlear implant with microphone, processor, coil and lead wire. The electrode array is inserted into the inner ear (the illustration is reproduced with permission from MED-EL, Innsbruck, Austria)

The cochlear implant (CI) is the standard treatment for uni- and bilateral severe and profound sensorineural hearing loss, both in adults and children. Around 600,000 deaf individuals have already received cochlear implantations worldwide [1]. Audible signals are detected by a microphone, processed into an electrical signal and transmitted transcutaneous to an implanted receiver. The signal is decoded and delivered through an electrode array implanted into the scala tympani of the cochlea onto the auditory nerve (Fig. 1).

The whole frequency range of the acoustic signal is split into different frequency bands and allocated to the different electrode contacts, mimicking the physiological tonotopic organization of the cochlea [2]. As speech understanding with the CI has improved, indication criteria have been extended towards patients with loss of hearing in the high frequencies and residual low-frequency hearing. Thinner, shorter and more flexible atraumatic electrodes have been designed to match the recipient’s individual cochlear anatomy to preserve its residual hearing [3].

Even though the preservation of the residual hearing is possible, however, the preservation rates achieved are not satisfactory. Substantial acoustic hearing loss (mean threshold increase of > 30 dB) is reported in 13% of cochlear implant patients receiving electric-acoustic stimulation (EAS) [4]. A moderate or partial loss of hearing (15 to 30 dB mean hearing loss) is described in 50.1% of the total patient population (*n* = 22) [5], and minimal loss of residual hearing (mean threshold shift < 15 dB) has been reported, e.g. for 59% of patients 2 years after implantation [6] and for more than 43% of patients 1 year after implantation [7]. Suhling et al. report a median hearing loss of 15 to 32.5 dB; shorter electrode arrays result in a better hearing preservation rate than using longer arrays [8].

The insertion of the electrode array into the cochlea has been shown to cause trauma to inner ear structures such as the basilar membrane, osseous spiral lamina and modiolus, and cascading molecular effects such as inflammation and oxidative stress [9, 10].

The loss of residual hearing after cochlear implantation is, as age-related hearing loss and noise-induced hearing loss, closely linked to the oxidative stress which is based on the formation of reactive oxygen species (ROS) [11]. The free radical formation is responsible for several types of damage to biological molecules present in the cochlea, as well as for the development of several other human diseases. In view of this, it would be desirable to be able to inactivate or reduce the formation of these free radicals, for example, by the use of antioxidants, agents that can inhibit or reduce damage in cells caused by free radicals. Foods, especially fruits, vegetables and legumes, also contain antioxidants, such as vitamins C, E and A; chlorophyllin; flavonoids; carotenoids; curcumin; and others that can restrict the dissemination of chain reactions and free radical-induced damage. Evidence has accumulated indicating that dietary antioxidant supplementation has become a therapeutic strategy to prevent and/or to delay the risks of sensorineural hearing loss [12]. Experimental studies on the auditory system have demonstrated that antioxidants plus a vasodilator reduce both noise- and drug- (aminoglycoside)-induced inner ear pathology and hearing loss by > 75% [13, 14]. This formulation is β-carotene (converted in the body to vitamin A), ascorbic acid (vitamin C), trolox (vitamin E) and the vasodilator magnesium. Together (ACEMg), they are remarkably effective in vivo to treat age-related hearing loss [15], noise-induced hearing loss [13, 16] and gentamycin-induced hearing loss [14]. Several reports indicate that free radicals play a key role in electrode-induced cochlear damage [9, 17]. The goal of ACEMg treatment is to reduce one important contributing factor, the stress of surgery and implantation of a foreign body that may negatively affect residual acoustic hearing in the implanted patient.

A meta-analysis aimed to evaluate randomized clinical trials to determine the effect of antioxidant supplementation on the auditory threshold in sensorineural hearing loss in patients of different age groups shows that there are no antioxidative therapies to preserve residual hearing after cochlear implantation [12].

The PROHEARING clinical trial (PROtect residual HEARING) is the first trial in humans to assess the efficacy of the antioxidative vitamins A, C and E and the vasodilator magnesium to reduce hearing loss caused by cochlear implantation in CI patients with residual hearing.

## Methods

This was a randomized, placebo-controlled, double-blind phase II clinical trial. The primary objective was to demonstrate that ACEMg is more efficacious than placebo in preserving residual hearing during cochlear implantation by comparing the hearing loss (air conduction hearing threshold postoperatively minus baseline threshold) at 500 Hz 3 months after the first fitting. For this purpose, cochlear implantation candidates with low-frequency residual hearing were recruited. Treatments (ACEMg or placebo) were administered orally for 106 days. Patients were followed up 12 months post first fitting which is approximately 13 months postoperatively. The study was registered (EudraCT-No.: 2012-005002-22), and as demanded by the National Institute for Health and Clinical Excellence for clinical research publications to allow greater transparency of the study methods and measures used [18], the detailed study protocol was published [19]. The study was performed according to the Declaration of Helsinki (in its current version) and other applicable national legislation. The study was approved by the competent authority (BfArM), and the ethics committee of Hannover Medical School issued a favourable opinion.

### Subjects

Study candidates were aged 18 years and older and suffered from pure/isolated cochlear-related hearing loss. This was proven with an objective audiological test battery before implantation. They had no or only little benefit from conventional hearing aids, defined as preoperative auditory speech understanding of less or equal to 60% in Freiburger monosyllables at 65 dB sound pressure level (SPL), best aided in the ear to be implanted. Their air conduction thresholds had to be better or equal than 85 dB hearing loss (HL) at 125 Hz, 90 dB HL at 250 Hz and better or equal than 95 dB HL at 500 Hz in the ear to be implanted. All patients received within routine CI surgery a Concerto or Synchrony implant from MED-EL (Innsbruck, Austria) (see the “Surgical technique and array choice” section).

### Sample size

The initial sample size calculation of this trial was based on historical data form the Hannover Medical School Department of Otolaryngology. In patients receiving different electrode lengths, a change in hearing thresholds at 500 Hz from baseline to 3 months after the first fitting (FF) of 15.6 dB with a standard deviation of 15.5 dB has been observed. Assuming equal standard deviations for ACEMg- and placebo-treated patients and a difference in change of hearing threshold between the treatment groups, the sample size calculation was conducted using a two-group *t* test with a two-sided type I error rate of 5%. Under these assumptions, 150 patients per treatment group were calculated to be necessary to achieve a power of 80%.

Due to recruitment difficulties caused by the inclusion and exclusion criteria, which may have been too stringent, the sample size target was reduced to 70 patients per group in a subsequent amendment resulting in a power of approximately 50% under the initial assumptions. However, for this phase II trial, the reduced sample size was judged acceptable to at least generate a signal whether ACEMg offers a promising treatment concept for the protection of residual hearing in CI patients [19].

### Blinding and randomization

Patients were randomized 1:1 to ACEMg or placebo. The randomization was stratified by the length of the electrodes (short (= 16 and 20 mm) vs. medium (24 mm) vs. long (28 mm) as planned preoperatively (albeit the length of the electrode was finally determined during surgery). Randomization was performed centrally using a web-based randomization tool. For details, please see [19].

### ACEMg treatment

All patients in the study received, per random allocation and double-blinded, treatment with ACEMg or placebo. The components of ACEMg are beta carotene (provitamine A, converted into retinol; 18 mg), vitamin C (magnesium ascorbate; 500 mg), vitamin E (dl-α-tocopherol acetate; 267 mg) and magnesium (magnesium citrate, magnesium ascorbate, magnesium stearate; 315 mg) (Table 1).

**Table 1:** ACEMg doses per tablet and per daily dose in milligrams

Component	Milligrams per tablet (label claim)	Milligrams per daily dose (6 tablets per day)
β-Carotene	3.0	18
Ascorbic acid	83.33	499.98
dl-α-tocopherol acetate	44.5	267
Magnesium	52.5	315

The patients received the daily dosage of ACEMg or placebo as 2 × 3 chewable tablets per day for a total of 106 days (first and last day 3 pills instead of 6) starting 2 days before CI surgery. The study medication was also taken preoperatively at the day of surgery and after surgery for 103 further days.

### Compliance

To quantify compliance with the study medication, serum vitamin E levels were measured in all randomized patients before, during (at surgery and 4 weeks postoperatively) and after the treatment period (month 3). Moreover, patients returned their treatment kits to the study site and provided information on their compliance via a short, self-administered questionnaire.

Patients under ACEMg treatment are only classified as compliant (and used for PP analysis), if their vitamin E level 4 weeks postoperatively was ≥ 20 μg/ml.

Patients in the placebo group with postoperative vitamin E levels ≥ 20 μg/ml were judged as non-compliant to the study procedures, since they agreed with their informed consent not to use daily multi-vitamins or other supplements during the course of the study, but show vitamin E levels that are unrealistic without vitamin supplements.

Additionally, patients were classified as non-compliant if they state in a questionnaire a non-compliance > 50%, and/or if the returned treatment kits contain > 50% of study medication, whereas missing bottles were considered as full bottles.

### Surgical technique and array choice

Surgery was performed by nine experienced otosurgeons at Hannover Medical School. The technique was described previously [20]. Based on individual levels of residual hearing, the length of the electrode array was chosen by the surgeon. Patients were implanted with a Flex28 (28 mm array length), Flex24 (24 mm array length), Flex20 (20 mm array length) or Flex16 (a custom-made 16-mm array) (all from MED-EL, Innsbruck, Austria). The Flex20 and Flex16 were grouped as “Flex20” for the analysis. All electrodes were inserted using the round window approach.

### Pure-tone audiometry

The primary objective was to compare the change in hearing thresholds at 500 Hz from baseline to 3 months after FF between the ACEMg- and placebo-treated groups.

To detect the frequency-specific acoustic hearing thresholds, air conduction thresholds were measured using a calibrated audiometer according to DIN EN 60318. The test method follows DIN ISO 8253 with headphones for air conduction and headset for bone conduction. Tested frequencies were 125, 250, 500 and 750 Hz, and 1, 2, 4 and 8 kHz. Pure-tone audiometer limits were 95 dB at 125 Hz, 100 dB at 250 Hz, and 110 dB at 500 to 1500 Hz. All subjects underwent pure-tone air conduction audiometry preoperatively, at the first activation and 3, 6 and 12 months after the first fitting.

To determine the frequency-specific hearing loss, the difference between the preoperative threshold level and the respective threshold measured at each postoperative time point was calculated.

### First activation

Four to 5 weeks after cochlear implantation, the audio processor OPUS 2 or DUET2, SONNET or SONNET EAS (MED-EL, Innsbruck, Austria) was first fitted in all patients.

### Data analysis

#### Analyses populations

The intention-to-treat (ITT) population consists of all randomized patients who took the study medication at least once. Patients were analysed as randomized independently of their real treatment (ACEMg or placebo).

The per-protocol (PP) population comprises all patients complying with the protocol, i.e. patients who were eligible, received the treatment they were randomized to, were compliant to the study medication (see above) and procedures and had an outcome assessment at the respective time points.

In the safety population, all patients were analysed who received at least one dose of the study medication (verum or placebo). Patients were analysed as treated.

#### Primary analysis

The primary analysis was performed in the ITT population and was conducted as an ANCOVA model (= primary analysis model) including the treatment (ACEMg or placebo), the baseline residual hearing at 500 Hz, the surgeon and the anticipated electrode length as covariates. The dependent variable was hearing loss at the implanted ear at 500 Hz 3 months post first fitting (hearing loss = 3 months post first fitting threshold minus 1–2 days preoperatively measured threshold) measured by air-conducted pure-tone audiometry. The frequency 500 Hz was chosen because it is the most important frequency for use of residual hearing, e.g. for voice recognition and speech in noise enhancement. If the patient reached at 500 Hz, the upper air-conducted detection limit of the audiometer (110 dB) without hearing, the measurement was set to 120 dB. Missing values were replaced by worst possible value per frequency (i.e. upper detection limit + 10 dB).

## Results

Based on the power calculation 140 patients (*n* = 70 verum and *n* = 70 Placebo) should have been enrolled [19]. The investigational medicinal products did run out of stability 18 months after project start. Since patients had to be supplied with study medication for 3 months, the actual total recruitment period was only 15 months. Due to financial insolvency of one of the consortium partners, resupply of study medication was not possible anymore and the trial had to be discontinued prematurely before the planned number of patients could be recruited. The authors believe that according to scientific standards it is necessary to publish data from discontinued clinical trials.

### Subjects

**Fig. 2:**
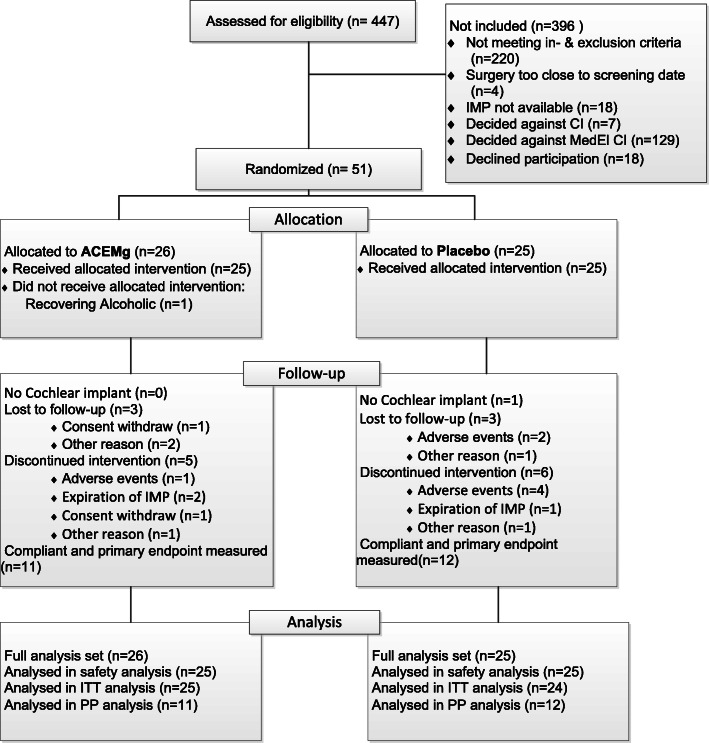
Patient flow chart

Four hundred forty-seven patients were accessed for eligibility. A total of *N* = 51 patients were enrolled until trial discontinuation, 26 in the ACEMg arm and 25 in the placebo arm. Figure 2 depicts in detail the reasons for not enrolling 396 patients. Two patients have been excluded from the efficacy analysis set (the ITT set): one from the placebo group because no cochlear implantation was conducted and one from the ACEMg group because he did not receive study medication. Therefore, *N* = 25 patients were analysed in the ACEMg group and *N* = 24 patients in the placebo group (both ITT set). Based on compliance measurements *N* = 11 (ACEMg) and *N* = 12 (placebo), patients were included in the per-protocol population (PP set) (Fig. 2).

The general medical history for all patients is stated in Additional file 1, supplement 1. Ear-specific medical history for the implanted ear is stated in Additional file 1, supplement 2. Supplement 3 shows the aetiology of the implanted ear. Concomitant medication for the ITT set summarized by events is stated in Additional file 1, supplement 4. Supplement 5 gives the hearing loss over time at 500 Hz with imputation of 110 dB if the upper detection limit is reached or 120 dB if the measurement is missing.

In Table 2, the baseline characteristics sex, age, implanted ear, electrode length and surgeon of the patients in the ITT set are stated by the treatment group. There are no clinically meaningful differences between the treatment groups. To investigate whether sex [21], age or baseline hearing at implantation did influence the treatment effect, a subgroup analysis was performed (see “Subgroup analysis” section).

**Table 2:** Baseline characteristics of the ITT set

	Placebo, *N* = 24	ACEMg, *N* = 25	Total, *N* = 49
Sex			
Male	9 (37.5%)	10 (40.0%)	19 (38.8%)
Female	15 (62.5%)	15 (60.0%)	30 (61.2%)
*p* value (*χ*^2^)			0.8575
Age			
*N*	24	25	49
Mean	57.38	53.64	55.47
STD	9.37	16.85	13.70
Mean difference			3.74
*p* value (*t* test)			0.3413
Treated ear			
Left	7 (29.2%)	11 (44.0%)	18 (36.7%)
Right	17 (70.8%)	14 (56.0%)	31 (63.3%)
*p* value (*χ*^2^)			0.2816
Used electrode length			
Flex 20	4 (16.7%)	3 (12.0%)	7 (14.3%)
Flex 24	4 (16.7%)	6 (24.0%)	10 (20.4%)
Flex 28	16 (66.7%)	16 (64.0%)	32 (65.3%)
*p* value (*χ*^2^)			0.7700
Surgeon			
Surgeon 1	2 (8.3%)	1 (4.0%)	3 (6.1%)
Surgeon 2	13 (54.2%)	16 (64.0%)	29 (59.2%)
Surgeon 3	2 (8.3%)	0 (0.0%)	2 (4.1%)
Surgeon 4	2 (8.3%)	3 (12.0%)	5 (10.2%)
Surgeon 7	1 (4.2%)	2 (8.0%)	3 (6.1%)
Surgeon 9	1 (4.2%)	0 (0.0%)	1 (2.0%)
Surgeon 10	1 (4.2%)	3 (12.0%)	4 (8.2%)
Surgeon 11	1 (4.2%)	0 (0.0%)	1 (2.0%)
Surgeon 12	1 (4.2%)	0 (0.0%)	1 (2.0%)
*p* value (*χ*^2^)			0.5195
Surgeon (categorized)			
Surgeon 2	13 (54.2%)	16 (64.0%)	29 (59.2%)
Other surgeons	11 (45.8%)	9 (36.0%)	20 (40.8%)
*p* value (*χ*^2^)			0.4839

### Hearing loss at 500 Hz 3 months after the first fitting

The primary efficacy parameter was hearing loss at the implanted ear at 500 Hz 3 months post first fitting (hearing loss = 3 months post first fitting threshold minus 1–2 days preoperatively measured threshold) measured by air-conducted pure-tone audiometry in the ear with a cochlear implant.

In the placebo group, the mean hearing loss (mean ± SD) was increased by 30.21 (± 15.84) dB whereas in the ACEMg group, the mean hearing loss increase was 26.00 (± 17.56) dB. The superiority of ACEMg over placebo in preserving residual hearing during cochlear implantation could not be concluded because the 95% confidence interval for the difference in hearing loss between the ACEMg and placebo groups derived from the ANCOVA model used for primary analysis (− 12.95, 4.65) includes zero (*p* = 0.3468, Table 3). Nevertheless, the estimate for ACEMg vs. placebo, adjusted for variables as listed in the ANCOVA model description, shows that descriptively, the hearing loss in the ACEMg group was on average 4.15 dB lower than in the placebo-treated patients (Table 3).

**Table 3:** Hearing loss (dB) 3 months post first fitting compared to baseline: results of the primary analysis ANCOVA model in the ITT set

	Estimate	Standard error	*p* value	95% confidence interval
ACEMg - placebo	− 4.15	4.3641140	0.3468	(− 12.95, 4.65)
Surgeon 2 - other surgeons	− 2.03	4.7141858	0.6688	(− 11.54, 7.48)
Flex 24 - Flex 20	35.32	9.9561928	0.0010	(15.24, 55.40)
Flex 28 - Flex 20	42.14	12.2218146	0.0013	(17.49, 66.79)
Flex 28 - Flex 24	6.82	6.8177036	0.3231	(− 6.93, 20.56)
Baseline hearing at 500 Hz (dB)	− 0.48	0.1849977	0.0136	(− 0.85, − 0.10)

**Fig. 3:**
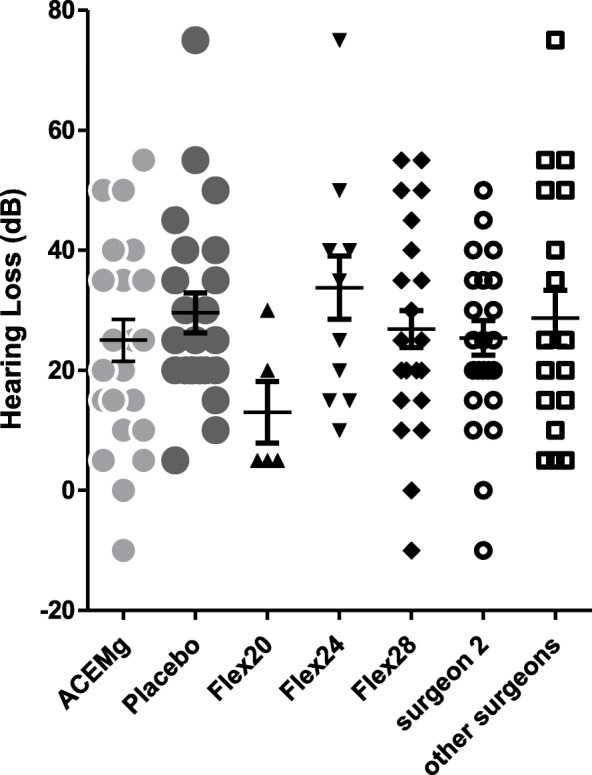
Hearing loss 3 months after the first fitting in patient groups receiving either ACEMg or placebo, grouped for the implanted electrode length (Flex 20 (= 16 mm and 20 mm), Flex 24 or Flex 28) and being implanted by surgeon 2 or other surgeons. No difference between ACEMg and placebo was observed, but patients receiving shorter electrodes showed less loss of residual hearing than those being implanted with a longer array

Figure 3 shows the individual hearing loss 3 months after the first fitting at 500 Hz by the treatment group, electrode length group and surgeon group.

Focusing on the used electrode lengths, it is obvious that the postoperative hearing loss (mean ± SD) of 11.25 ± 12.50 dB in individuals implanted with a Flex 20 or Flex 16 (*n* = 4) is tremendously lower than in patients receiving a Flex 24 (*n* = 13; 32.69 ± 17.87 dB) or a Flex 28 (*n* = 32; 28.28 ± 15.74 dB). Statistical analyses revealed a significantly lower loss of residual hearing in Flex 20-implanted patients compared to those receiving a Flex 24 or 28 (Table 3). The effect estimates for the different electrode lengths and the effect estimate for an overall effect of electrode length indicate that electrode length and hearing loss are not independent (ANCOVA derived *p* value for an overall effect of electrode length: *p* = 0.0029). The shorter the used electrode, the smaller is the hearing loss in this study. However, as secondary findings, these results are merely exploratory and do not allow causal inference because electrode length was not randomly assigned, but chosen preoperative by the surgeon. Out of the 49 patients receiving a CI, 29 were implanted by one surgeon (surgeon 2) and 20 patients were implanted by 8 other surgeons (Table 2 and Fig. 3). Since these 8 surgeons conducted at maximum 5 surgeries each, they were clustered for the statistical analysis. No statistically significant difference in hearing loss after surgery was detected between the patients based on surgeon (*p* value = 0.66) even though patients being implanted by surgeon 2 exhibited a 2.03-dB lower mean hearing loss than patients being implanted by the other surgeons (Table 3).

### Sensitivity analyses of primary analysis

**Fig. 4:**
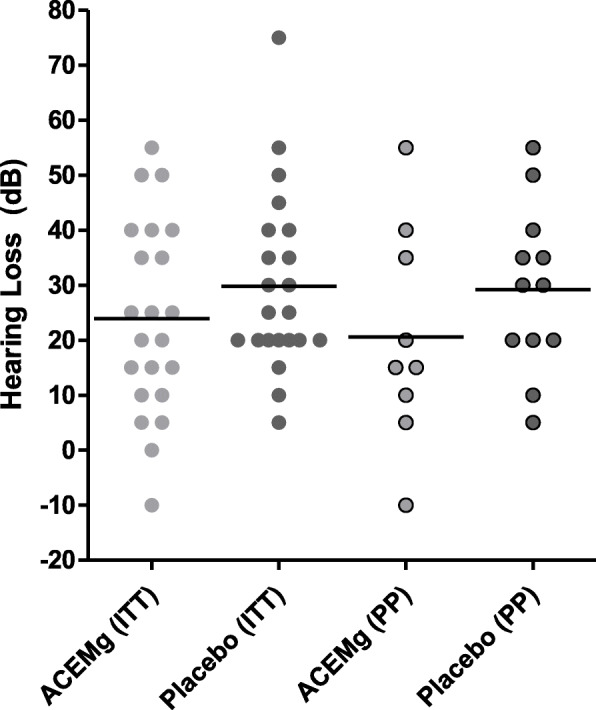
Individual hearing loss 3 months after the first fitting in ACEMg- or placebo-treated patients of the ITT and PP population

As a pre-defined sensitivity analysis, the primary analysis model (conducted as an ANCOVA model as described in the “Primary analysis” section) was applied in the PP population. Table 4 states the descriptive analysis of the primary endpoint in the PP population. In this population, the mean ± SD hearing loss in the placebo group was 29.17 (± 15.05) dB and in the ACEMg group 23.64 (± 18.99) dB. Here, the median hearing loss differed from 30 dB in the placebo-treated patients to 20 dB in the ACEMg group. Table 5 gives the results of the primary analysis model in the PP population. All sensitivity analyses are in line with the primary analysis result of the ITT population. The estimates for the treatment effect favour ACEMg over placebo without statistical significance and, thus, supporting the overall conclusion, that no superiority of ACMEg over placebo could be demonstrated in this prematurely determined study (Fig. 4). In the PP population, however, on average, the placebo-treated patients have 8.01 dB more hearing loss than ACEMg-treated patients (Table 5), which is a clinically relevant difference.

**Table 4:** Hearing loss (dB) 3 months post first fitting compared to baseline: a descriptive analysis of the primary endpoint—PP set

Subgroup	Number	Mean	Std. Dev.	Minimum	Median	Maximum
Placebo	12	29.17	15.05	5.00	30.00	55.00
ACEMg	11	23.64	18.99	− 10.00	20.00	55.00
Flex 20	3	13.33	14.43	5.00	5.00	30.00
Flex 24	6	28.33	15.71	10.00	27.50	50.00
Flex 28	14	28.57	17.59	− 10.00	32.50	55.00
Surgeon 2	16	25.94	14.97	− 10.00	30.00	50.00
Other surgeons	7	27.86	21.96	5.00	20.00	55.00

**Table 5:** Hearing loss (dB) 3 months post first fitting compared to baseline: results of the analysis of the primary endpoint—PP set

	Estimate	Standard error	*p* value	95% confidence interval
ACEMg - placebo	− 8.01	7.4323051	0.2961	(− 23.69, 7.67)
Surgeon 2 - other surgeon	− 1.29	9.7399943	0.8963	(− 21.84, 19.26)
Flex 24 - Flex 20	26.40	15.9253500	0.1157	(− 7.20, 60.00)
Flex 28 - Flex 20	32.73	19.8918623	0.1182	(− 9.23, 74.70)
Flex 28 - Flex 24	6.33	10.5704737	0.5569	(− 15.97, 28.64)
Baseline	− 0.28	0.3292886	0.4037	(− 0.98, 0.41)

### Subgroup analysis

Subgroup analyses using the primary analysis model have been conducted by sex, age and baseline hearing measured by air-conducted audiometry at 500 Hz (Table 6). Regarding age, the study population was divided by the overall median age into two groups: age is (I) younger or equal to 55 years or (II) older than 55 years.

**Table 6:** Hearing loss 3 months post first fitting compared to baseline: a descriptive subgroup analysis of the primary endpoint by age and residual hearing—ITT set

Baseline factor	Subgroup	Treatment	Number	Mean HL	Std. Dev.
Age	Younger or equal to 55 years	Placebo	11	35.91	17.72
		ACEMg	15	21.33	17.97
	Older than 55 years	Placebo	13	25.38	12.82
		ACEMg	10	33.00	15.13
Residual hearing at 500 Hz	Less or equal to 65 dB	Placebo	9	37.22	21.23
		ACEMg	8	24.38	18.41
	More than 65 dB	Placebo	15	26.00	10.21
		ACEMg	17	26.76	17.67

Regarding baseline hearing measured by air-conducted audiometry at 500 Hz, the study population was divided by hearing threshold of 65 dB into two groups: hearing at 500 Hz is (i) less or equal to 65 dB or (ii) larger than 65 dB.

Subgroup results regarding sex are in line with the primary analysis results (data not shown). The treatment effect estimates in both subgroups did not differ significantly from 0. Thus, the primary analysis results interpretation did not change, and no inferential conclusions could be made.

The treatment effect estimates for ACEMg vs. placebo in the young and old subgroup were contrary. While in the subgroup of patients of less or equal to 55 years, ACEMg is estimated to be superior to placebo (8.24 dB lower hearing loss in ACEMg than in placebo), in the subgroup of patients older than 55 years, placebo is estimated to be superior over ACEMg (4.56 dB lower hearing loss in placebo than in ACEMg). However, both estimates did not differ significantly from 0 (Table 7).

**Table 7:** Hearing loss 3 months post first fitting compared to baseline: results of the subgroup analysis by age—ITT set

Subgroup	Effect	Estimate	Standard error	*p* value	95% confidence interval
Younger or equal to 55 years	ACEMg - placebo	− 8.24	7.7076232	0.2980	(− 24.31, 7.84)
Older than 55 years	ACEMg - placebo	4.56	6.5936026	0.4981	(− 9.35, 18.48)

Adjusting the primary analysis model for age (either as a continuous variable or a binary variable) did not change the analysis results regarding the treatment effect, and the estimated interaction term does not differ significantly from 0 (see Table 8 for continuous adjustment). Thus, the primary analysis results interpretation did not change, and no inferential conclusions could be made.

**Table 8:** Hearing loss 3 months post first fitting compared to baseline: effect estimate for ACEMg vs. placebo, surgeon, electrode length and age from ANCOVA model adjusted by age as a continuous covariate

Effect	Estimate	Standard error	*p* value	95% confidence interval
ACEMg - placebo	− 3.14	4.3410215	0.4735	(− 11.90, 5.62)
Surgeon 2 - other surgeons	− 2.65	4.6540781	0.5715	(− 12.05, 6.74)
Flex 24 - Flex 20	33.99	9.8300736	0.0013	(14.15, 53.83)
Flex 28 - Flex 20	35.54	12.7415605	0.0079	(9.82, 61.25)
Flex 28 - Flex 24	1.55	7.5051096	0.8378	(− 13.60, 16.69)
Baseline	− 0.37	0.1935072	0.0605	(− 0.76, 0.02)
Age	0.28	0.1782778	0.1254	(− 0.08; 0.64)

Subgroup analyses regarding baseline hearing at 500 Hz are given in Table 9 descriptively, and the results are given in Table 10. Subgroup analyses regarding baseline hearing show a smaller hearing loss in the ACEMg group in both subgroups. Additionally, a larger treatment effect can be observed in the subgroup with a baseline hearing threshold of less or equal to 65 dB. However, in neither subgroup, the treatment difference is statistically significant. Subgroup results regarding baseline hearing are in line with the primary analysis. Nevertheless, it has to be mentioned that in this dataset, the effect of age (years) on hearing loss at 500 Hz is estimated to be an increased hearing loss of 0.28 dB for each year of age at which the surgery is conducted (Table 8).

**Table 9:** Hearing loss 3 months post first fitting compared to baseline: descriptive subgroup analysis by baseline hearing at 500 Hz air-conducted audiometry (> 65 dB or ≤ 65 dB)—ITT set

Hearing loss 3 months post-fitting compared to baseline—ITT set							
Subgroup	Treatment	*N*	Mean	Std. Dev.	Minimum	Median	Maximum
Less or equal to 65 dB	Placebo	9	37.22	21.23	5.00	40.00	75.00
	ACEMg	8	24.38	18.41	5.00	17.50	55.00
More than 65 dB	Placebo	15	26.00	10.21	10.00	25.00	45.00
	ACEMg	17	26.76	17.67	− 10.00	25.00	50.00

**Table 10:** Hearing loss 3 months post first fitting compared to baseline: results of ANCOVA model applied for subgroup analyses by baseline hearing at 500 Hz air-conducted audiometry (> 65 dB or ≤ 65 dB)—ITT set

Subgroup	Effect	Estimate	Standard error	*p* value	95% confidence interval
Less or equal to 65 dB	ACEMg - placebo	− 11.46	9.1677502	0.2374	(− 31.63, 8.72)
	Surgeon 2 - other surgeons	− 3.12	9.6611576	0.7529	(− 24.38, 18.15)
	Flex 24 - Flex 20	23.26	13.8967484	0.1224	(− 7.33, 53.85)
	Flex 28 - Flex 20	27.69	17.8852439	0.1498	(− 11.68, 67.06)
	Flex 28 - Flex 24	4.43	12.9790722	0.7393	(− 24.14, 33.00)
More than 65 dB	ACEMg - placebo	− 1.89	4.4766332	0.6767	(− 11.07, 7.30)
	Surgeon 2 - other surgeons	− 0.23	4.8327054	0.9623	(− 10.15, 9.69)
	Flex 28 - Flex 24	13.09	8.2152402	0.1227	(− 3.77, 29.95)

### Hearing loss over time

**Fig. 5:**
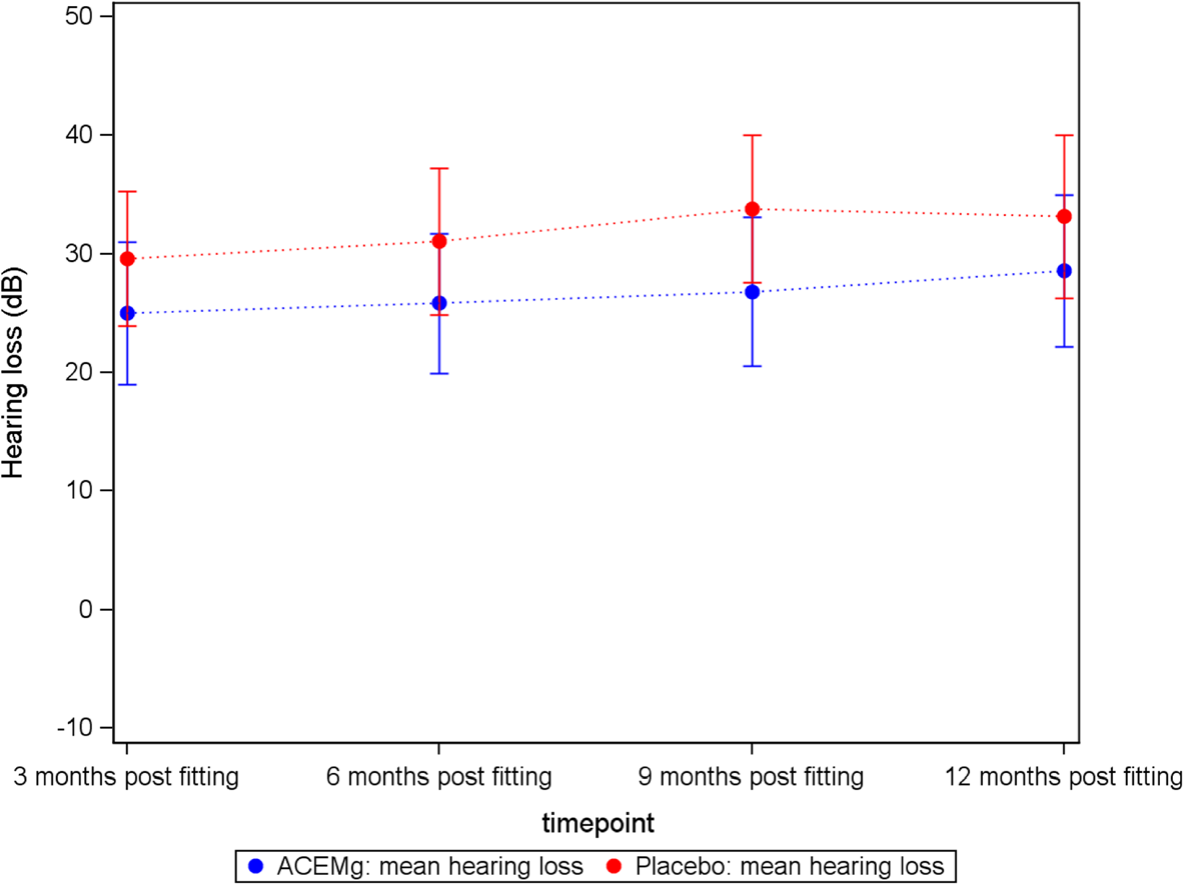
Mean hearing loss over time in the ACEMg- and placebo-treated groups. The calculation is based on the available measurements at each time point applying the following imputation: If the upper detection limit is reached (110 dB), 120 dB are imputed. Missing values not due to measurement limits are not imputed. At all observed time points, the hearing loss in the placebo group was higher compared to the hearing loss detected in the ACEMg group. In both groups, the hearing loss increased over time

The mean hearing loss increased over time in ACEMg- and placebo-treated patients from 25.00 respectively 29.57 dB 3 months after the first fitting to 28.57 respectively 33.16 dB 12 months after first fitting (Table 11, Fig. 5), resulting in a difference (ACEMg − placebo) of − 4.59 dB. Over time, the number of patients conducting the respective study visits declined. In the ACEMg group, of the 25 patients being implanted (Fig. 2), 21 did finish their 12-month appointment. In the placebo group, from 24 implanted patients, only 19 conducted their 12-months visit (Table 11). When imputing 120 dB for missing measurements, as was conducted in the primary analysis only regarding hearing loss 3 months post first fitting, the difference (ACEMg − placebo) in hearing loss between ACEMg and placebo 12 months after the first fitting of 29.80 dB − 36.25 dB is − 6.45 dB (Additional file 1, supplement 5). As an additional sensitivity analysis, all time points were analysed simultaneously with a mixed model for repeated measures accounting for the correlation between the measurements of the same patient. In contrast to the analysis of the separate time points, here, the difference between ACEMg and placebo (ACEMg − placebo) 12 months post first fitting is estimated to be − 1.1 (*p* = 0.79), which indicates no perseverance of the observed difference 3 months post first fitting (see Additional file 1, supplement 6 for a short model description and respective results). This result conflicts with the former results analysing the individual time points separately and applying different strategies for handling missing values (excluding missing values or imputing 120 dB, respectively). Consequently, the high amount of missing values for the measurements 12 months post first fitting in the ITT set (19%) and the above-described dependence of the estimated difference between ACEMg and placebo 12 months post first fitting on the imputation method used for missing values do not allow robust conclusions on the course and persistence of the treatment effect.

**Table 11:** Hearing loss at 500 Hz at all time points compared to baseline

Time point	Treatment	Number	Mean	Std. Dev.	Minimum	Median	Maximum
3 months post-fitting	Placebo	23	29.57	15.88	5.00	25.00	75.00
	ACEMg	24	25.00	17.19	− 10.00	25.00	55.00
6 months post-fitting	Placebo	24	31.04	17.69	5.00	27.50	75.00
	ACEMg	24	25.83	16.79	− 5.00	25.00	55.00
9 months post-fitting	Placebo	21	33.81	16.50	15.00	30.00	75.00
	ACEMg	22	26.82	17.08	− 5.00	25.00	55.00
12 months post-fitting	Placebo	19	33.16	17.34	5.00	30.00	75.00
	ACEMg	21	28.57	16.89	5.00	25.00	55.00

The calculation is based on the available measurements at each time point applying the following imputation: If the upper detection limit is reached (110 dB), 120 dB is imputed. Missing values not due to measurement limits are not imputed

### Safety

In total, 29 adverse events (AE) were registered, 14 of which were serious (supplement 7). Most adverse events were of grade 1 (68.9%). 10.3% and 17.2% were of grades 2 and 3, respectively. One event (3.4%) was of grade 4. No grade 5 adverse events were documented. Patients recovered completely from most AEs (*n* = 26, 89.7%). The outcome of two AEs was recovered with a sequel (hypertension and upper limb fracture) while the status of one AE remained unknown at database closure.

The number of both non-serious and serious adverse events (SAE) was numerically higher in the placebo group (*n* = 18 for AEs, *n* = 11 for SAEs) than in the ACEMg group (*n* = 11 and *n* = 3 for AEs and SAEs, respectively).

Also, the number of AEs that resulted in the withdrawal of the study medication was numerically higher in the placebo group (*n* = 15) than in the ACEMg group (*n* = 8). Finally, the number of AEs that were assessed as possibly causally related to the study medication (*n* = 4: ear pain, tympanic membrane perforation, vertigo and cerebrospinal fluid leakage) by the reporter was also higher in the placebo group (*n* = 10) than in the verum group (*n* = 4).

## Discussion

### Efficacy

Most recently, Wanna et al. detected hearing preservation rates of 38% 2–3 weeks postoperatively decreasing to 18% at about 19 months post-surgery [22]. Since many variables may affect the residual hearing directly during and after cochlear implantation as well as months and years after surgery, the reason for hearing loss may be multifactorial. Electrode design, surgical approach, and inflammation have all been implicated as potentially important variables.

In this study, we focused on one possible aspect leading to post-implantation hearing loss. We concentrated on the free radical formation and the possible reduction using antioxidative vitamins and a vasodilator to increase cochlear blood flow. In this placebo-controlled single-centre clinical trial, the effect of orally administered vitamins A, C and E together with magnesium on residual hearing in CI patients and the dependency of hearing loss after cochlear implantation on age at implantation, residual hearing status, sex or surgeon was evaluated.

As described above, the study did not reach the recruitment goal due to IMP-resupply mismanagement of one partner, resulting in 51 included patients (= 36%) of the planned sample size of 140.

Nevertheless, the analysis of the primary objective in the recruited ITT population showed that the mean hearing loss measured at 500 Hz 3 months post first fitting compared to baseline in the placebo group was 30.21 (± 15.84) dB whereas in the ACEMg group, the hearing loss was 26.00 (± 17.56) dB. There was no statistically significant difference, but a 4.15-dB smaller mean hearing loss was observed in the ACEMg-treated patients compared to the placebo group. Clinically, in general, a difference of 4.15 dB in hearing ability is a relevant improvement. This tendency of residual hearing preservation 3 months after the first fitting was still detectable 1 year after the implantation with 36.25 dB mean hearing loss in patients receiving placebo and 29.80 dB mean hearing loss in ACEMg-treated patients, even though the main amount of radicals is released immediately after implantation. This suggests that the initial intake of ACEMg within the first 3 months after the first fitting may have a prolonged protective effect on residual hearing. As in the placebo group, the hearing loss was ongoing in the ACEMg group within the first 12 months after the first fitting. For the ACEMg group, some analysis suggested a lower extent of hearing loss over time than the loss over time detected in the placebo group. However, it needs to be noted that for 18% of the patients in the ITT set, the measurement of residual hearing 12 months after the first fitting was missing, and the prolonged protective effect could not be replicated in sensitivity analyses applying different missing data assumptions. Consequently, further studies have to be conducted to proof this potential benefit of ACEMg.

Since the likelihood of residual hearing preservation in cochlear implantation is related to medical history and surgical factors such as duration of hearing loss, aetiology, route of implantation (round window approach vs. cochleostomy), and device-related factors such as shape, length and flexibility of the array, it has to be concluded that there are multiple factors which affect the residual hearing. Zanetti et al. correlated age, side of implant, implant model and type of cochleostomy to hearing loss after cochlear implantation and reported that the mean threshold variations did not reach statistical significance for any of these variables. A slight trend in favour of better residual hearing preservation in children vs. adults was seen, especially at lower frequencies [23]. Conform to Zanetti’s results, we did not find a dependency of hearing loss and ear to be implanted (data not shown). But there was a strong dependency of hearing preservation and electrode length used with smaller hearing loss for shorter electrodes and higher loss for longer ones. This is in accordance with other studies reporting good hearing preservation when using shorter electrodes [8].

In the placebo group, the hearing loss at 500 Hz determined 3 months (30.21 ± 15.84 dB) or 12 months (36.25 ± 19.63 dB) after the first fitting was in the range of hearing preservation rates reported previously [4, 8]. We detected a not significant tendency of progressive hearing loss over time in placebo-treated patients, which was already reported by others [6, 24–26].

In our study, the subjects enrolled did not substantially differ regarding their demographical data between the treatment groups. The mean age in the ACEMg group was 3.74 years lower than in the placebo group (*p* = 0.3413). Clinically, 3.7 years are judged as irrelevant, but since the residual hearing may correlate with age due to age-related hearing loss, the hearing performance of the patients may decline with age. Therefore age is a relevant factor and it has to be avoided to have a mismatch in age between the treatment groups. The potential influence of this imbalance was investigated by subgroup analyses with age added as either continuous or dichotomized (by median age) factor to the primary analysis model. Without showing statistically significant differences, the subgroup analyses estimate ACEMg to be superior to placebo in patients aged 55 years or younger with an effect size of 8.24 dB lesser hearing loss in ACEMg-treated patients. This effect is turned to the opposite in patient older than 55 years, where placebo shows a not significantly reduced hearing loss with an estimated effect of 4.56 dB. It is well known that, in general, the free radical formation is higher in older people [27]. We hypothesize that the additional oxidative stress due to the electrode insertion [9] may lead to a summating of free radicals resulting in an oxidative stress overload in older patients which cannot be treated by the ACEMg doses regiment used in our study.

### Safety

The number and spectrum of AEs fall within the expected range for the population studied. Also, the number of AEs that were likely to be related to the study procedure is to be expected in the context of the surgical procedure. No increased risk compared to placebo could be observed in ACEMg-treated patients. ACEMg was generally well-tolerated with AE frequencies at (or below) the placebo level. No actions for safety had to be taken during the PROHEARING trial.

## Conclusion

The data of this prematurely discontinued trial show a tendency that ACEMg therapy in patients aged 55 years and younger starting directly before cochlear implantation and lasting for about 100 days may lead to a better hearing preservation 3 months after the first fitting and lasting for at least 13 months after surgery. We hypothesize that a food supplement of the metabolic imbalances during and after surgery by antioxidative and vasodilatative supplementation may improve the benefit some patients will have by receiving a CI. Further studies need to be conducted addressing this topic to verify the promising preliminary data of this trial.

## Supplementary information

**Additional file 1.** Includes the general medical history for all patients in the ITT-set as Supplement 1. Ear specific medical history for the implanted ear is stated in Supplement 2 of the additional file 1. Supplement 3 shows the aetiology of the implanted ear. Concomitant medication for the ITT-set summarized by events is stated in Supplement 4. Supplement 5 gives the hearing loss over time at 500 Hz with imputation of 110 dB if the upper detection limit is reached or 120 dB if the measurement is missing. Supplement 6 reports the hearing preservation measured by air conducted audiometry at 500 Hz including all timepoints analyzed in the modified ITT population using a mixed model with change from baseline as dependent variable and treatment, baseline hearing threshold, surgeon, electrode length, electrode length - visit interaction and visit-treatment interaction as fixed effects. The mixed model for repeated measures was conducted adjusting for either the planned electrode length (Supplement Table 6.1) as was done in the primary analysis model, or by adjusting for the used electrode length (Supplement Table 6.2). The estimated mean hearing loss at all time points with 95%-confidence intervals based on the mixed model for repeated measures adjusted for the planned electrode length is illustrated in supplementary figure 6. AEs are listed in supplement 7.

## Data Availability

The datasets used and analysed during the current study are available from the corresponding author on reasonable request.

## Abbreviations

ACEMg: Vitamins A, C and E and magnesium
AE: Adverse event
CI: Cochlear implant
EAS: Electric-acoustic stimulation
FF: First fitting
HL: Hearing loss
ITT: Intention-to-treat
MAX: Maximum
PP: Per-protocol
ROS: Reactive oxygen species
SAE: Serious adverse event
SD: Standard deviation
SPL: Sound pressure level
