# Supplementary material for: Randomized placebo-controlled clinical trial investigating the effect of antioxidants and a vasodilator on overall safety and residual hearing preservation in cochlear implant patients
Source: Trials. 2020 Jul 14;21:643. doi: 10.1186/s13063-020-04522-9 (PMC7362557; doi:10.1186/s13063-020-04522-9)
Supplement: Supplementary file 1 — Additional file 1. Includes the general medical history for all patients in the ITT-set as Supplement 1. Ear specific medical history for the implanted ear is stated in Supplement 2 of the additional file 1. Supplement 3 shows the aetiology of the implanted ear. Concomitant medication for the ITT-set summarized by events is stated in Supplement 4. Supplement 5 gives the hearing loss over time at 500 Hz with imputation of 110 dB if the upper detection limit is reached or 120 dB if the measurement is missing. Supplement 6 reports the hearing preservation measured by air conducted audiometry at 500 Hz including all timepoints analyzed in the modified ITT population using a mixed model with change from baseline as dependent variable and treatment, baseline hearing threshold, surgeon, electrode length, electrode length - visit interaction and visit-treatment interaction as fixed effects. The mixed model for repeated measures was conducted adjusting for either the planned electrode length (Supplement Table 6.1) as was done in the primary analysis model, or by adjusting for the used electrode length (Supplement Table 6.2). The estimated mean hearing loss at all time points with 95%-confidence intervals based on the mixed model for repeated measures adjusted for the planned electrode length is illustrated in supplementary figure 6. AEs are listed in supplement 7. [file 13063_2020_4522_MOESM1_ESM.docx]

Supplement 1: General medical history - ITT-set

| **Analysis set: ITT-set** | | | |
| --- | --- | --- | --- |
|  | **Treatment** | | |
|  | **Placebo N=24** | **ACEMg N=25** | **Total N=49** |
| **Rotatory Vertigo** | | | |
| no | 14 ( 58.3%) | 21 ( 84.0%) | 35 ( 71.4%) |
| yes | 10 ( 41.7%) | 4 ( 16.0%) | 14 ( 28.6%) |
| p-VALUE (CHI2) |  |  | 0.0468 |
| **Familiar deafness** | | | |
| no | 16 ( 66.7%) | 15 ( 60.0%) | 31 ( 63.3%) |
| yes | 8 ( 33.3%) | 10 ( 40.0%) | 18 ( 36.7%) |
| p-VALUE (CHI2) |  |  | 0.6284 |
| **Infectious disease** | | | |
| no | 23 ( 95.8%) | 25 (100.0%) | 48 ( 98.0%) |
| yes | 1 ( 4.2%) | 0 ( 0.0%) | 1 ( 2.0%) |
| p-VALUE (CHI2) |  |  | 0.3024 |
| **Renal disease** | | | |
| no | 23 ( 95.8%) | 25 (100.0%) | 48 ( 98.0%) |
| yes | 1 ( 4.2%) | 0 ( 0.0%) | 1 ( 2.0%) |
| p-VALUE (CHI2) |  |  | 0.3024 |
| **Cervical spine disease** | | | |
| no | 14 ( 58.3%) | 16 ( 64.0%) | 30 ( 61.2%) |
| yes | 10 ( 41.7%) | 9 ( 36.0%) | 19 ( 38.8%) |
| p-VALUE (CHI2) |  |  | 0.6840 |
| **Thyroid disease** | | | |
| no | 19 ( 79.2%) | 16 ( 64.0%) | 35 ( 71.4%) |
| yes | 5 ( 20.8%) | 8 ( 32.0%) | 13 ( 26.5%) |
| unknown | 0 ( 0.0%) | 1 ( 4.0%) | 1 ( 2.0%) |
| p-VALUE (CHI2) |  |  | 0.3810 |
| **Lactose intolerance** | | | |
| no | 22 ( 91.7%) | 21 ( 84.0%) | 43 ( 87.8%) |
| yes | 1 ( 4.2%) | 2 ( 8.0%) | 3 ( 6.1%) |
| unknown | 1 ( 4.2%) | 2 ( 8.0%) | 3 ( 6.1%) |
| p-VALUE (CHI2) |  |  | 0.7154 |

Supplement 2: Medical history in implanted ear - ITT-set

| **Analysis set: ITT-set** | | | |
| --- | --- | --- | --- |
|  | **Treatment** | | |
|  | **Placebo N=24** | **ACEMg N=25** | **Total N=49** |
| **Years since hearing loss in implanted ear** | | | |
| N | 24 | 24 | 48 |
| MISSING | 0 | 1 | 1 |
| MEAN | 21.13 | 19.79 | 20.46 |
| STD | 12.71 | 10.62 | 11.61 |
| MIN | 1 | 1 | 1 |
| MEDIAN | 20 | 20.50 | 20 |
| MAX | 55 | 39 | 55 |
| 95% CI MEAN | [15.76 ; 26.49] | [15.31 ; 24.28] | [17.09 ; 23.83] |
| MEAN DIFFERENCE |  |  | 1.33 |
| 95% CI MEAN DIFFERENCE |  |  | [-5.47 ; 8.14] |
| p-VALUE (T-TEST) |  |  | 0.6952 |
| **Years since hearing loss in implanted ear (categorized)** | | | |
| Missing | 0 | 1 | 1 |
| 0-5 years | 1 ( 4.2%) | 3 ( 12.5%) | 4 ( 8.3%) |
| 6-10 years | 1 ( 4.2%) | 4 ( 16.7%) | 5 ( 10.4%) |
| 11-15 years | 8 ( 33.3%) | 0 ( 0.0%) | 8 ( 16.7%) |
| 16-20 years | 3 ( 12.5%) | 5 ( 20.8%) | 8 ( 16.7%) |
| 21-25 years | 7 ( 29.2%) | 4 ( 16.7%) | 11 ( 22.9%) |
| 26-30 years | 0 ( 0.0%) | 6 ( 25.0%) | 6 ( 12.5%) |
| 31-40 years | 2 ( 8.3%) | 2 ( 8.3%) | 4 ( 8.3%) |
| 51-60 years | 2 ( 8.3%) | 0 ( 0.0%) | 2 ( 4.2%) |
| p-VALUE (CHI2) |  |  | 0.0053 |
| **Severity of hearing loss (implanted ear)** | | | |
| progressive | 21 ( 87.5%) | 19 ( 76.0%) | 40 ( 81.6%) |
| sudden | 1 ( 4.2%) | 5 ( 20.0%) | 6 ( 12.2%) |
| unknown | 2 ( 8.3%) | 1 ( 4.0%) | 3 ( 6.1%) |
| p-VALUE (CHI2) |  |  | 0.2143 |
| **Hearing aid (implanted ear)** | | | |
| no | 6 ( 25.0%) | 6 ( 24.0%) | 12 ( 24.5%) |
| yes | 18 ( 75.0%) | 19 ( 76.0%) | 37 ( 75.5%) |
| p-VALUE (CHI2) |  |  | 0.9351 |
| **Years since hearing aid in implanted ear** | | | |
| N | 18 | 19 | 37 |
| MISSING | 6 | 6 | 12 |
| MEAN | 18.72 | 17.89 | 18.30 |
| STD | 12.77 | 10.52 | 11.51 |
| MIN | 6 | 1 | 1 |
| MEDIAN | 15 | 18 | 17 |
| MAX | 49 | 37 | 49 |
| 95% CI MEAN | [12.37 ; 25.07] | [12.82 ; 22.97] | [14.46 ; 22.14] |
| MEAN DIFFERENCE |  |  | 0.83 |
| 95% CI MEAN DIFFERENCE |  |  | [-6.96 ; 8.62] |
| p-VALUE (T-TEST) |  |  | 0.8305 |
| **Years since hearing aid in implanted ear (categorized)** | | | |
| not applicable | 6 ( 25.0%) | 6 ( 24.0%) | 12 ( 24.5%) |
| 0-5 years | 0 ( 0.0%) | 1 ( 4.0%) | 1 ( 2.0%) |
| 6-10 years | 6 ( 25.0%) | 6 ( 24.0%) | 12 ( 24.5%) |
| 11-15 years | 3 ( 12.5%) | 2 ( 8.0%) | 5 ( 10.2%) |
| 16-20 years | 3 ( 12.5%) | 1 ( 4.0%) | 4 ( 8.2%) |
| 21-25 years | 3 ( 12.5%) | 3 ( 12.0%) | 6 ( 12.2%) |
| 26-30 years | 0 ( 0.0%) | 4 ( 16.0%) | 4 ( 8.2%) |
| 31-40 years | 1 ( 4.2%) | 2 ( 8.0%) | 3 ( 6.1%) |
| 41-50 years | 2 ( 8.3%) | 0 ( 0.0%) | 2 ( 4.1%) |
| p-VALUE (CHI2) |  |  | 0.3847 |
| **Tinnitus (implanted ear)** | | | |
| no | 9 ( 37.5%) | 8 ( 32.0%) | 17 ( 34.7%) |
| yes | 14 ( 58.3%) | 15 ( 60.0%) | 29 ( 59.2%) |
| sometimes | 1 ( 4.2%) | 2 ( 8.0%) | 3 ( 6.1%) |
| p-VALUE (CHI2) |  |  | 0.8161 |
| **Years since tinnitus in implanted ear** | | | |
| N | 13 | 15 | 28 |
| MISSING | 11 | 10 | 21 |
| MEAN | 12.31 | 13.47 | 12.93 |
| STD | 10.29 | 9.54 | 9.73 |
| MIN | 1 | 1 | 1 |
| MEDIAN | 12 | 12 | 12 |
| MAX | 38 | 29 | 38 |
| 95% CI MEAN | [6.09 ; 18.53] | [8.18 ; 18.75] | [9.16 ; 16.70] |
| MEAN DIFFERENCE |  |  | -1.16 |
| 95% CI MEAN DIFFERENCE |  |  | [-8.86 ; 6.55] |
| p-VALUE (T-TEST) |  |  | 0.7597 |
| **Years since tinnitus in implanted ear (categorized)** | | | |
| Missing | 2 | 2 | 4 |
| not applicable | 9 ( 40.9%) | 8 ( 34.8%) | 17 ( 37.8%) |
| 0-5 years | 4 ( 18.2%) | 4 ( 17.4%) | 8 ( 17.8%) |
| 6-10 years | 2 ( 9.1%) | 3 ( 13.0%) | 5 ( 11.1%) |
| 11-15 years | 3 ( 13.6%) | 1 ( 4.3%) | 4 ( 8.9%) |
| 16-20 years | 2 ( 9.1%) | 4 ( 17.4%) | 6 ( 13.3%) |
| 21-25 years | 1 ( 4.5%) | 1 ( 4.3%) | 2 ( 4.4%) |
| 26-30 years | 0 ( 0.0%) | 2 ( 8.7%) | 2 ( 4.4%) |
| 31-40 years | 1 ( 4.5%) | 0 ( 0.0%) | 1 ( 2.2%) |
| p-VALUE (CHI2) |  |  | 0.6715 |
| **If tinnitus, intensity (implanted ear)** | | | |
| N | 15 | 17 | 32 |
| MISSING | 9 | 8 | 17 |
| MEAN | 5.87 | 5.12 | 5.47 |
| STD | 2.53 | 2.71 | 2.61 |
| MIN | 2 | 2 | 2 |
| MEDIAN | 5 | 5 | 5 |
| MAX | 10 | 10 | 10 |
| 95% CI MEAN | [4.46 ; 7.27] | [3.72 ; 6.51] | [4.53 ; 6.41] |
| MEAN DIFFERENCE |  |  | 0.75 |
| 95% CI MEAN DIFFERENCE |  |  | [-1.15 ; 2.65] |
| p-VALUE (T-TEST) |  |  | 0.4277 |
| **Previous ear operations (implanted ear)** | | | |
| no | 20 ( 83.3%) | 22 ( 88.0%) | 42 ( 85.7%) |
| yes | 4 ( 16.7%) | 3 ( 12.0%) | 7 ( 14.3%) |
| p-VALUE (CHI2) |  |  | 0.6407 |
| **Years since operation in implanted ear** | | | |
| N | 4 | 3 | 7 |
| MISSING | 20 | 22 | 42 |
| MEAN | 11.25 | 25.67 | 17.43 |
| STD | 9.91 | 18.23 | 14.81 |
| MIN | 2 | 6 | 2 |
| MEDIAN | 9.50 | 29 | 14 |
| MAX | 24 | 42 | 42 |
| 95% CI MEAN | [-4.52 ; 27.02] | [-19.62 ; 70.95] | [3.73 ; 31.12] |
| MEAN DIFFERENCE |  |  | -14.42 |
| 95% CI MEAN DIFFERENCE |  |  | [-41.61 ; 12.78] |
| p-VALUE (T-TEST) |  |  | 0.2312 |
| **Years since operation in implanted ear (categorized)** | | | |
| not applicable | 20 ( 83.3%) | 22 ( 88.0%) | 42 ( 85.7%) |
| 0-5 years | 2 ( 8.3%) | 0 ( 0.0%) | 2 ( 4.1%) |
| 6-10 years | 0 ( 0.0%) | 1 ( 4.0%) | 1 ( 2.0%) |
| 11-15 years | 1 ( 4.2%) | 0 ( 0.0%) | 1 ( 2.0%) |
| 21-25 years | 1 ( 4.2%) | 0 ( 0.0%) | 1 ( 2.0%) |
| 26-30 years | 0 ( 0.0%) | 1 ( 4.0%) | 1 ( 2.0%) |
| 41-50 years | 0 ( 0.0%) | 1 ( 4.0%) | 1 ( 2.0%) |
| p-VALUE (CHI2) |  |  | 0.3137 |
| **If previous ear operation, what operation? (implanted ear)** | | | |
| MISSING | 20 | 22 | 42 |
| Cholesteatom | 1 ( 25.0%) | 0 ( 0.0%) | 1 ( 14.3%) |
| MET (2009), 2. OP 2011: Revision | 1 ( 25.0%) | 0 ( 0.0%) | 1 ( 14.3%) |
| Tympanoskopie | 1 ( 25.0%) | 0 ( 0.0%) | 1 ( 14.3%) |
| Tympanometry and sealing of round and oval window | 1 ( 25.0%) | 0 ( 0.0%) | 1 ( 14.3%) |
| stapesplastic | 0 ( 0.0%) | 1 ( 33.3%) | 1 ( 14.3%) |
| tympanostomy tube | 0 ( 0.0%) | 2 ( 66.7%) | 2 ( 28.6%) |
| p-VALUE (CHI2) |  |  | 0.2206 |

Supplement 3: Aetiology of implanted ear - ITT-set

| **Analysis set: ITT-set** | | | |
| --- | --- | --- | --- |
|  | **Treatment** | | |
|  | **Placebo N=24** | **ACEMg N=25** | **Total N=49** |
| **Congenital** | | | |
| not applicable | 23 ( 95.8%) | 24 ( 96.0%) | 47 ( 95.9%) |
| yes | 1 ( 4.2%) | 1 ( 4.0%) | 2 ( 4.1%) |
| **Trauma** | | | |
| not applicable | 22 ( 91.7%) | 22 ( 88.0%) | 44 ( 89.8%) |
| yes | 2 ( 8.3%) | 3 ( 12.0%) | 5 ( 10.2%) |
| **Unknown** | | | |
| MISSING | 9 | 11 | 20 |
| yes | 15 (100.0%) | 14 (100.0%) | 29 (100.0%) |
| **Other** | | | |
| MISSING | 18 | 17 | 35 |
| yes | 6 (100.0%) | 8 (100.0%) | 14 (100.0%) |
| **If other, specification** | | | |
| MISSING | 18 | 17 | 35 |
| Circulatory disorder of the inner ear | 0 ( 0.0%) | 1 ( 12.5%) | 1 ( 7.1%) |
| Hearing Loss (probably because of stress) | 0 ( 0.0%) | 1 ( 12.5%) | 1 ( 7.1%) |
| Hearing Loss and Stress | 0 ( 0.0%) | 1 ( 12.5%) | 1 ( 7.1%) |
| Morbus Meniere is conjectured | 1 ( 16.7%) | 0 ( 0.0%) | 1 ( 7.1%) |
| Noise + Family ( mother , father , grandmother hard of hearing ) | 1 ( 16.7%) | 0 ( 0.0%) | 1 ( 7.1%) |
| Usher-Syndrom | 0 ( 0.0%) | 1 ( 12.5%) | 1 ( 7.1%) |
| acute hearing loss | 1 ( 16.7%) | 0 ( 0.0%) | 1 ( 7.1%) |
| inherited and presbyacusis | 0 ( 0.0%) | 1 ( 12.5%) | 1 ( 7.1%) |
| noise and stress | 1 ( 16.7%) | 0 ( 0.0%) | 1 ( 7.1%) |
| otitis of the middle ear | 0 ( 0.0%) | 1 ( 12.5%) | 1 ( 7.1%) |
| preterm birth | 1 ( 16.7%) | 0 ( 0.0%) | 1 ( 7.1%) |
| psychic | 0 ( 0.0%) | 1 ( 12.5%) | 1 ( 7.1%) |
| sudden hearing loss +poor blood circulation | 1 ( 16.7%) | 0 ( 0.0%) | 1 ( 7.1%) |
| under vasculitic | 0 ( 0.0%) | 1 ( 12.5%) | 1 ( 7.1%) |

Supplement 4: Concomitant medication by events - ITT-set

|  | **Treatment** | |
| --- | --- | --- |
| **Medication Indication** | **Placebo  N=24** | **ACEMg  N=25** |
| Total number of concomitant medications | 108 | 106 |
| ASS | - | 1 ( 0.9%) |
| headache | - | 1 ( 0.9%) |
| ASS 100 | - | 3 ( 2.8%) |
| apoplectic stroke | - | 1 ( 0.9%) |
| blood thinners | - | 1 ( 0.9%) |
| unknown | - | 1 ( 0.9%) |
| ASS Protect 100 mg | 1 ( 0.9%) | - |
| because anoyrisma | 1 ( 0.9%) | - |
| Aciclostad 800mg | 1 ( 0.9%) | - |
| herpes zoster | 1 ( 0.9%) | - |
| Allopurinol | 2 ( 1.9%) | - |
| high uric acid | 1 ( 0.9%) | - |
| unknown | 1 ( 0.9%) | - |
| Amlodipin | 1 ( 0.9%) | 1 ( 0.9%) |
| high blood pressure | 1 ( 0.9%) | - |
| hypertension | - | 1 ( 0.9%) |
| Antibiotic | - | 1 ( 0.9%) |
| sore throat | - | 1 ( 0.9%) |
| Antibiotic | - | 1 ( 0.9%) |
| earache | - | 1 ( 0.9%) |
| Antibiotic | 1 ( 0.9%) | - |
| sinuses suppurated | 1 ( 0.9%) | - |
| Arcoxia 60 mg | 1 ( 0.9%) | - |
| only temporarily during a gout flare | 1 ( 0.9%) | - |
| Arlevert | - | 1 ( 0.9%) |
| vertigo | - | 1 ( 0.9%) |
| Artelac Nighttime Gel | 1 ( 0.9%) | - |
| dry eyes | 1 ( 0.9%) | - |
| Atacand | 1 ( 0.9%) | - |
| hypertension | 1 ( 0.9%) | - |
| Atenolol | - | 1 ( 0.9%) |
| blood pressure | - | 1 ( 0.9%) |
| Atosil 40ml | - | 1 ( 0.9%) |
| sleep problems | - | 1 ( 0.9%) |
| Augmentan 875/125mg | - | 1 ( 0.9%) |
| wound healing disorder | - | 1 ( 0.9%) |
| Baldriparan | - | 1 ( 0.9%) |
| insomnia | - | 1 ( 0.9%) |
| Berotec | 1 ( 0.9%) | - |
| asthma | 1 ( 0.9%) | - |
| Beta Blocker | 1 ( 0.9%) | - |
| heart arrhythmia | 1 ( 0.9%) | - |
| Betahistin | - | 1 ( 0.9%) |
| vertigo | - | 1 ( 0.9%) |
| Bisohexal | 1 ( 0.9%) | - |
| unknown | 1 ( 0.9%) | - |
| Bisolich | 1 ( 0.9%) | - |
| hypertension | 1 ( 0.9%) | - |
| Bisoprolol | 1 ( 0.9%) | 2 ( 1.9%) |
| unknown | - | 1 ( 0.9%) |
| blood pressure | - | 1 ( 0.9%) |
| hypertension | 1 ( 0.9%) | - |
| blood pressure tablets | 1 ( 0.9%) | - |
| high blood pressure | 1 ( 0.9%) | - |
| Calcium | 1 ( 0.9%) | - |
| systremma | 1 ( 0.9%) | - |
| Candecor 16mg | 1 ( 0.9%) | - |
| hypertension | 1 ( 0.9%) | - |
| Candesartan | 1 ( 0.9%) | 1 ( 0.9%) |
| high blood pressure | - | 1 ( 0.9%) |
| hypertension | 1 ( 0.9%) | - |
| Cefuroxim 500 | 1 ( 0.9%) | - |
| antibiotic after CI-OP | 1 ( 0.9%) | - |
| Chlorprothixen-Neurax 50mg | 1 ( 0.9%) | - |
| depression | 1 ( 0.9%) | - |
| Chlorprothixen-neuraxpharm 15mg | 1 ( 0.9%) | - |
| depression | 1 ( 0.9%) | - |
| Cidox | 1 ( 0.9%) | - |
| CI-OP | 1 ( 0.9%) | - |
| Citalopram | 2 ( 1.9%) | - |
| depression, panic attacks | 1 ( 0.9%) | - |
| psyche | 1 ( 0.9%) | - |
| Clopidogrel 75mg | - | 1 ( 0.9%) |
| for stent with coating | - | 1 ( 0.9%) |
| Clopidrogel | 1 ( 0.9%) | - |
| because of apoplexy | 1 ( 0.9%) | - |
| Codein | 1 ( 0.9%) | - |
| common cold | 1 ( 0.9%) | - |
| Codiovan | 1 ( 0.9%) | - |
| blood pressure | 1 ( 0.9%) | - |
| Corifeo Lercanidipinhydrochlorid | 1 ( 0.9%) | - |
| hypertension | 1 ( 0.9%) | - |
| Cortison | - | 1 ( 0.9%) |
| UNK | - | 1 ( 0.9%) |
| Cough suppressants | 1 ( 0.9%) | - |
| cough | 1 ( 0.9%) | - |
| Cymbalta | 1 ( 0.9%) | - |
| UNK | 1 ( 0.9%) | - |
| Debutamin | - | 1 ( 0.9%) |
| for examination with stress echo (heart coronary arteries) | - | 1 ( 0.9%) |
| Decortin H | 1 ( 0.9%) | - |
| acute hearing loss | 1 ( 0.9%) | - |
| Dekristol | 1 ( 0.9%) | - |
| UNK | 1 ( 0.9%) | - |
| Diazepam | 1 ( 0.9%) | - |
| depression, panic attacks | 1 ( 0.9%) | - |
| Diclofenac | 1 ( 0.9%) | - |
| ache | 1 ( 0.9%) | - |
| Dolormin | 1 ( 0.9%) | - |
| menstrual pain | 1 ( 0.9%) | - |
| Dominal Forte | - | 1 ( 0.9%) |
| sleep problems | - | 1 ( 0.9%) |
| Doxepin - neuraxpharm 40mg | - | 1 ( 0.9%) |
| depression | - | 1 ( 0.9%) |
| Doxicilin | - | 1 ( 0.9%) |
| Pelvic inflammatory disease | - | 1 ( 0.9%) |
| Doxycyclin 200 A 2 | - | 1 ( 0.9%) |
| peritonitis | - | 1 ( 0.9%) |
| Eardrops | 1 ( 0.9%) | - |
| Otitis media (not implanted side) | 1 ( 0.9%) | - |
| Eferox | - | 1 ( 0.9%) |
| unknown | - | 1 ( 0.9%) |
| Enalapril | - | 1 ( 0.9%) |
| blood pressure | - | 1 ( 0.9%) |
| Eva Kadin 75 | 1 ( 0.9%) | - |
| strong period | 1 ( 0.9%) | - |
| Ferro-sanol | - | 1 ( 0.9%) |
| hypoferremia | - | 1 ( 0.9%) |
| Ferrosanol 40mg | - | 1 ( 0.9%) |
| hypoferremia | - | 1 ( 0.9%) |
| Fluvastatin Actavis | 1 ( 0.9%) | - |
| to reduce cholesterol | 1 ( 0.9%) | - |
| Folate 15 mg | - | 1 ( 0.9%) |
| for side effects of MTX | - | 1 ( 0.9%) |
| Foster 100/6 | - | 1 ( 0.9%) |
| asthma | - | 1 ( 0.9%) |
| Furosemid | - | 1 ( 0.9%) |
| to much water in the body | - | 1 ( 0.9%) |
| Gelomyrtol | - | 1 ( 0.9%) |
| infection/common cold | - | 1 ( 0.9%) |
| HYLO-COMOD | 1 ( 0.9%) | - |
| eye dryness | 1 ( 0.9%) | - |
| Herz ASS 100 | 1 ( 0.9%) | - |
| heart arrhythmia | 1 ( 0.9%) | - |
| Hetformin | 1 ( 0.9%) | - |
| diabetes | 1 ( 0.9%) | - |
| Hustensaft | - | 1 ( 0.9%) |
| infection/common cold | - | 1 ( 0.9%) |
| Hylo Comod Drops | 1 ( 0.9%) | - |
| dry eyes | 1 ( 0.9%) | - |
| Hylo-Vision Gel sine | 1 ( 0.9%) | - |
| eye dryness | 1 ( 0.9%) | - |
| Hypnorex retard | 1 ( 0.9%) | - |
| depression | 1 ( 0.9%) | - |
| IVIG ICE 200g | - | 1 ( 0.9%) |
| paraproteinemic sensorymotoriy PNP with paraproteinemia Type IGA Lambda | - | 1 ( 0.9%) |
| Ibu 600 | - | 1 ( 0.9%) |
| sprained hock | - | 1 ( 0.9%) |
| Ibuflam | - | 1 ( 0.9%) |
| UNK | - | 1 ( 0.9%) |
| Ibuprofen | 2 ( 1.9%) | - |
| headache | 1 ( 0.9%) | - |
| common cold | 1 ( 0.9%) | - |
| Ibuprofen 600 | - | 1 ( 0.9%) |
| sprained ankle | - | 1 ( 0.9%) |
| Influenza vaccination | 1 ( 0.9%) | - |
| influenza prevention | 1 ( 0.9%) | - |
| Insulin | 1 ( 0.9%) | - |
| diabetes | 1 ( 0.9%) | - |
| Insulin | - | 1 ( 0.9%) |
| diabetes | - | 1 ( 0.9%) |
| Insulin: Humalog | 1 ( 0.9%) | - |
| diabetes | 1 ( 0.9%) | - |
| Insulin: Huminsulin basal 19020 | 1 ( 0.9%) | - |
| diabetes | 1 ( 0.9%) | - |
| Iodid | 1 ( 0.9%) | 1 ( 0.9%) |
| thyroid disorder | 1 ( 0.9%) | - |
| thyroid hypofunction | - | 1 ( 0.9%) |
| Charcoal tablet | 1 ( 0.9%) | - |
| diarrhoea | 1 ( 0.9%) | - |
| L- Thyrox Hexal 200mcg | 1 ( 0.9%) | - |
| thyroid disorder | 1 ( 0.9%) | - |
| L-Thyroxin | 2 ( 1.9%) | 3 ( 2.8%) |
| hypothyroidism | 1 ( 0.9%) | - |
| thyroid disfunction | 1 ( 0.9%) | - |
| thyroid drug | - | 1 ( 0.9%) |
| thyroid hypofunction | - | 1 ( 0.9%) |
| unknown | - | 1 ( 0.9%) |
| L-Thyroxin 150 mg | - | 1 ( 0.9%) |
| hypothyroidism/thyroid harvesting | - | 1 ( 0.9%) |
| L-Thyroxin 75mg | - | 1 ( 0.9%) |
| thyroid hypofunction | - | 1 ( 0.9%) |
| L-Thyroxin Henning 50 plus | - | 1 ( 0.9%) |
| unknown | - | 1 ( 0.9%) |
| Lercanidipin | 1 ( 0.9%) | - |
| hypertension | 1 ( 0.9%) | - |
| Lora ADGC | - | 1 ( 0.9%) |
| allergy | - | 1 ( 0.9%) |
| Lozzar | 1 ( 0.9%) | - |
| high blood pressure | 1 ( 0.9%) | - |
| Lyrika | - | 1 ( 0.9%) |
| earache | - | 1 ( 0.9%) |
| MTX | - | 1 ( 0.9%) |
| rheumatism | - | 1 ( 0.9%) |
| MTX 15 mg | - | 1 ( 0.9%) |
| vasculitis | - | 1 ( 0.9%) |
| Macrogol | 1 ( 0.9%) | - |
| constipation | 1 ( 0.9%) | - |
| Magnesium | - | 1 ( 0.9%) |
| muscle cramps | - | 1 ( 0.9%) |
| Metformin | 2 ( 1.9%) | - |
| diabetes | 2 ( 1.9%) | - |
| Metformin 1000 | - | 2 ( 1.9%) |
| diabetes | - | 2 ( 1.9%) |
| Meto Succinat Sandoz | 1 ( 0.9%) | - |
| hypertension | 1 ( 0.9%) | - |
| MetoHexal | - | 1 ( 0.9%) |
| high blood pressure | - | 1 ( 0.9%) |
| Metoprolol | 1 ( 0.9%) | 1 ( 0.9%) |
| unknown | 1 ( 0.9%) | 1 ( 0.9%) |
| Micardis | 1 ( 0.9%) | - |
| hypertension | 1 ( 0.9%) | - |
| Mictonorm | 1 ( 0.9%) | - |
| unknown | 1 ( 0.9%) | - |
| Mirtazapin | - | 1 ( 0.9%) |
| depression and short of sleep | - | 1 ( 0.9%) |
| Mirtazapin 30mg | - | 1 ( 0.9%) |
| against insomnia | - | 1 ( 0.9%) |
| Mirtazapin Al 15mg | 1 ( 0.9%) | - |
| depression | 1 ( 0.9%) | - |
| Mydocalm | - | 1 ( 0.9%) |
| rheumatism | - | 1 ( 0.9%) |
| Neupro Pflaster | 1 ( 0.9%) | - |
| Parkinson | 1 ( 0.9%) | - |
| Novalgin | 1 ( 0.9%) | - |
| ache | 1 ( 0.9%) | - |
| Novaminsulfon | - | 1 ( 0.9%) |
| pain | - | 1 ( 0.9%) |
| Oekolp Ovula | - | 1 ( 0.9%) |
| ailment, because of estrogen deficiency | - | 1 ( 0.9%) |
| Ear dropsTri/Aq. pu 50% | - | 1 ( 0.9%) |
| recommendation after CI-surgery | - | 1 ( 0.9%) |
| Omeprazol | 2 ( 1.9%) | 3 ( 2.8%) |
| gastric protection | 1 ( 0.9%) | 1 ( 0.9%) |
| gastroenteritis | - | 1 ( 0.9%) |
| stomach pain because of Ibu | - | 1 ( 0.9%) |
| stomach protection | 1 ( 0.9%) | - |
| Oton | 1 ( 0.9%) | - |
| hardening in the neck | 1 ( 0.9%) | - |
| Otriven | - | 1 ( 0.9%) |
| recommendation after CI-surgery | - | 1 ( 0.9%) |
| Panotile | - | 1 ( 0.9%) |
| left otitis externa | - | 1 ( 0.9%) |
| Pantoprazol | 1 ( 0.9%) | 1 ( 0.9%) |
| protection of the stomach | 1 ( 0.9%) | - |
| UNK | - | 1 ( 0.9%) |
| Pantozol 40 mg | - | 1 ( 0.9%) |
| gastric protection due to the other medicines | - | 1 ( 0.9%) |
| Pentoxifyllin | 1 ( 0.9%) | - |
| sudden hearing loss (left side) | 1 ( 0.9%) | - |
| Prastatin 40 mg | 1 ( 0.9%) | - |
| because of apoplexy | 1 ( 0.9%) | - |
| Prednisolon | 1 ( 0.9%) | 2 ( 1.9%) |
| Vertigo after OP | - | 1 ( 0.9%) |
| rheumatism | - | 1 ( 0.9%) |
| sudden hearing loss (left side) | 1 ( 0.9%) | - |
| Prednisolon 10mg | - | 1 ( 0.9%) |
| lumbar pain | - | 1 ( 0.9%) |
| Prednisolon 250mg - 100mg | 1 ( 0.9%) | - |
| acute hearing loss | 1 ( 0.9%) | - |
| Prednisolon 5 mg/ Since 01.06.2015 7,5 mg | - | 1 ( 0.9%) |
| cortison for vasculitis | - | 1 ( 0.9%) |
| Prednisolon acis 10mg | 1 ( 0.9%) | - |
| tinnitus | 1 ( 0.9%) | - |
| Ramilich | 1 ( 0.9%) | 1 ( 0.9%) |
| high blood pressure | - | 1 ( 0.9%) |
| hypertension | 1 ( 0.9%) | - |
| Ramipril | 1 ( 0.9%) | 3 ( 2.8%) |
| hypertension | - | 1 ( 0.9%) |
| blood pressure | - | 1 ( 0.9%) |
| high blood pressure | - | 1 ( 0.9%) |
| hypertension | 1 ( 0.9%) | - |
| Ramipril 2,5mg | - | 1 ( 0.9%) |
| for high blood pressure | - | 1 ( 0.9%) |
| Ramipril-1A-Harma | - | 1 ( 0.9%) |
| UNK | - | 1 ( 0.9%) |
| Ramipril-Isis | 1 ( 0.9%) | - |
| high blood pressure | 1 ( 0.9%) | - |
| Ranexa 375mg | - | 1 ( 0.9%) |
| prevention of angina pectoris | - | 1 ( 0.9%) |
| Repaglinid | 1 ( 0.9%) | - |
| diabetes | 1 ( 0.9%) | - |
| Roceptin | 2 ( 1.9%) | - |
| liquorleck | 2 ( 1.9%) | - |
| Salbutamol | 1 ( 0.9%) | - |
| asthma | 1 ( 0.9%) | - |
| Simbicort | - | 1 ( 0.9%) |
| chronic bronchitis | - | 1 ( 0.9%) |
| Simva | - | 1 ( 0.9%) |
| cholesterol reducing | - | 1 ( 0.9%) |
| Simvastatin | 2 ( 1.9%) | 3 ( 2.8%) |
| apoplectic stroke | - | 1 ( 0.9%) |
| cholesterol levels | - | 1 ( 0.9%) |
| cholesterol value too high | 1 ( 0.9%) | - |
| cholesterol-lowering drugs | 1 ( 0.9%) | - |
| to high cholesterol | - | 1 ( 0.9%) |
| Sinnpret | - | 1 ( 0.9%) |
| infection/common cold | - | 1 ( 0.9%) |
| Sinupret | 1 ( 0.9%) | - |
| cold | 1 ( 0.9%) | - |
| Sinupret ACC | 1 ( 0.9%) | - |
| common cold | 1 ( 0.9%) | - |
| Sinvastatin | 1 ( 0.9%) | - |
| diabetes | 1 ( 0.9%) | - |
| Spiriva | - | 1 ( 0.9%) |
| chronic brinchitis | - | 1 ( 0.9%) |
| Sulpirid 200 | - | 1 ( 0.9%) |
| UNK | - | 1 ( 0.9%) |
| Symbiocort | 1 ( 0.9%) | - |
| bronchial asthma | 1 ( 0.9%) | - |
| Symbiose | - | 1 ( 0.9%) |
| earache | - | 1 ( 0.9%) |
| Tamulsin | 1 ( 0.9%) | - |
| prostate enlargement | 1 ( 0.9%) | - |
| Tansipret | - | 1 ( 0.9%) |
| infection/common cold | - | 1 ( 0.9%) |
| Thealoz Duo | 1 ( 0.9%) | - |
| dry eyes | 1 ( 0.9%) | - |
| Theophyllin | - | 1 ( 0.9%) |
| asthma | - | 1 ( 0.9%) |
| Thyronajod | 2 ( 1.9%) | 1 ( 0.9%) |
| thyroid | - | 1 ( 0.9%) |
| thyroid disfunction | 1 ( 0.9%) | - |
| unknown | 1 ( 0.9%) | - |
| Timo Comod 0,5% | - | 1 ( 0.9%) |
| glaucoma | - | 1 ( 0.9%) |
| Titalokram | - | 1 ( 0.9%) |
| depression | - | 1 ( 0.9%) |
| Torasemid | - | 1 ( 0.9%) |
| high blood pressure | - | 1 ( 0.9%) |
| Traumeel | 1 ( 0.9%) | - |
| to support the healing process | 1 ( 0.9%) | - |
| Trental | 2 ( 1.9%) | 1 ( 0.9%) |
| tinnitus | 1 ( 0.9%) | - |
| vertigo | 1 ( 0.9%) | - |
| tinnitus | - | 1 ( 0.9%) |
| Tromcardin | 1 ( 0.9%) | - |
| cardiac arrythmia | 1 ( 0.9%) | - |
| UNK | 1 ( 0.9%) | - |
| neuralgia | 1 ( 0.9%) | - |
| Unazink | - | 1 ( 0.9%) |
| infection/common cold | - | 1 ( 0.9%) |
| Valdoxan 25mg | - | 1 ( 0.9%) |
| depression | - | 1 ( 0.9%) |
| Valsartan | - | 1 ( 0.9%) |
| high blood pressure | - | 1 ( 0.9%) |
| Valsatan | - | 1 ( 0.9%) |
| hypertension | - | 1 ( 0.9%) |
| Vigantoletten | 1 ( 0.9%) | 1 ( 0.9%) |
| osteoporosis | 1 ( 0.9%) | - |
| prevention of osteoporosis | - | 1 ( 0.9%) |
| Vit. B12 | - | 1 ( 0.9%) |
| Vit. B12 defency | - | 1 ( 0.9%) |
| Vitamin D3 3000 | - | 1 ( 0.9%) |
| disorder of the immune system psoriasis | - | 1 ( 0.9%) |
| Xailin Gel | 1 ( 0.9%) | - |
| dry eyes | 1 ( 0.9%) | - |
| Xipamid | 1 ( 0.9%) | - |
| hypertension | 1 ( 0.9%) | - |
| Zinktabletten | 1 ( 0.9%) | - |
| immune system debilitated | 1 ( 0.9%) | - |
| Zopiclon | 1 ( 0.9%) | - |
| insomnia | 1 ( 0.9%) | - |
| Zopiclon 7,5 | - | 1 ( 0.9%) |
| UNK | - | 1 ( 0.9%) |
| antibiotic | 1 ( 0.9%) | 2 ( 1.9%) |
| infection in neck and throat, temperature | 1 ( 0.9%) | - |
| bronchitis | - | 1 ( 0.9%) |
| long-lasting cough | - | 1 ( 0.9%) |
| antibiotics | 1 ( 0.9%) | - |
| quincy and extraction of a tooth | 1 ( 0.9%) | - |
| baldrian | 1 ( 0.9%) | - |
| sleeping problems | 1 ( 0.9%) | - |
| no comment | 2 ( 1.9%) | 2 ( 1.9%) |
| UNK | 2 ( 1.9%) | 2 ( 1.9%) |
| **Table includes all concomitant medications. Percentages are calculated using the total number of  concomitant medications per treatment group as the denominator.** | | |

Supplement 5: Hearing loss at 500 Hz at all time points compared to baseline - ITT set

| **Timepoint** | **treatment** | **N** | **Mean** | **Std. Dev.** | **Minimum** | **Median** | **Maximum** |
| --- | --- | --- | --- | --- | --- | --- | --- |
| 3 months post fitting | Placebo | 24 | 30.21 | 15.84 | 5.00 | 25.00 | 75.00 |
|  | ACEMg | 25 | 26.00 | 17.56 | -10.00 | 25.00 | 55.00 |
| 6 months post fitting | Placebo | 24 | 31.04 | 17.69 | 5.00 | 27.50 | 75.00 |
|  | ACEMg | 25 | 26.80 | 17.13 | -5.00 | 25.00 | 55.00 |
| 9 months post fitting | Placebo | 24 | 36.67 | 19.37 | 15.00 | 30.00 | 85.00 |
|  | ACEMg | 25 | 28.80 | 17.03 | -5.00 | 30.00 | 55.00 |
| 12 months post fitting | Placebo | 24 | 36.25 | 19.63 | 5.00 | 32.50 | 85.00 |
|  | ACEMg | 25 | 29.80 | 16.10 | 5.00 | 35.00 | 55.00 |
| The calculation is based on the available measurements at each timepoint applying the following imputation: If upper detection limit is reached (110dB) or the measurement is missing, 120dB are imputed. | | | | | | | |

Supplement 6

Hearing preservation measured by air conducted audiometry at 500 Hz including all timepoints was analyzed in the modified ITT population using a mixed model with change from baseline as dependent variable and treatment, baseline hearing threshold, surgeon, electrode length, electrode length ‐ visit interaction and visit‐treatment interaction as fixed effects. Visit is modelled as repeated factor within patient with an unstructured covariance structure.

In the modified ITT population, 5 patients were excluded from the ITT analysis population due to missing baseline values.

The mixed model for repeated measures was conducted adjusting for either the planned electrode length as was done in the primary analysis model, or by adjusting for the used electrode length.

Table 6.1: Hearing loss at 500 Hz at all time points compared to baseline: Estimated means based on the mixed model for repeated measures – modified ITT set

| **Visit** | **Treatment group** | **Electrode length as covariate** | **Estimate (estimated mean)** | **Standard Error** |
| --- | --- | --- | --- | --- |
| intra-OP | Placebo | Planned electrode length | 31.7318 | 2.9885 |
|  |  | Used electrode length | 32.0430 | 3.1405 |
|  | ACEMg | Planned electrode length | 30.3794 | 2.9272 |
|  |  | Used electrode length | 30.0153 | 3.0747 |
| first-fitting | Placebo | Planned electrode length | 30.2628 | 2.8841 |
|  |  | Used electrode length | 30.5560 | 3.1713 |
|  | ACEMg | Planned electrode length | 27.5897 | 2.8249 |
|  |  | Used electrode length | 27.2428 | 3.1049 |
| 3 months post fitting | Placebo | Planned electrode length | 30.2802 | 3.1098 |
|  |  | Used electrode length | 30.7820 | 3.2196 |
|  | ACEMg | Planned electrode length | 26.1483 | 3.0588 |
|  |  | Used electrode length | 25.5748 | 3.1631 |
| 6 months post fitting | Placebo | Planned electrode length | 31.1518 | 3.1496 |
|  |  | Used electrode length | 31.7549 | 3.2134 |
|  | ACEMg | Planned electrode length | 26.8636 | 3.1072 |
|  |  | Used electrode length | 26.1787 | 3.1656 |
| 9 months post fitting | Placebo | Planned electrode length | 32.2892 | 3.0428 |
|  |  | Used electrode length | 33.0349 | 3.0929 |
|  | ACEMg | Planned electrode length | 27.6243 | 2.9941 |
|  |  | Used electrode length | 26.9368 | 3.0399 |
| 12 months post fitting | Placebo | Planned electrode length | 31.1786 | 2.9614 |
|  |  | Used electrode length | 32.0580 | 3.1422 |
|  | ACEMg | Planned electrode length | 30.0664 | 2.9022 |
|  |  | Used electrode length | 29.2920 | 3.0755 |

Table 6.2: Hearing loss at 500 Hz at all time points compared to baseline: Estimated treatment effects at all time points based on the mixed model for repeated measures – modified ITT set

| **Estimated effect** | **Electrode length as covariate** | **Estimate** | **Standard Error** | **Lower Limit of 95% Confidence Intervall** | **Upper Limit of 95% Confidence Intervall** | **P-value** |
| --- | --- | --- | --- | --- | --- | --- |
| ACEMg - Placebo | Planned electrode length | -3.0371 | 3.6713 | -10.4410 | 4.3667 | 0.4127 |
|  | Used electrode length | -4.1648 | 3.9194 | -12.0689 | 3.7394 | 0.2939 |
| ACEMg - Placebo post-operative | Planned electrode length | -1.3524 | 4.1907 | -9.8038 | 7.0990 | 0.7485 |
|  | Used electrode length | -2.0278 | 4.4150 | -10.9315 | 6.8760 | 0.6483 |
| ACEMg - Placebo at first fitting | Planned electrode length | -2.6731 | 4.0448 | -10.8302 | 5.4839 | 0.5122 |
|  | Used electrode length | -3.3131 | 4.4582 | -12.3039 | 5.6777 | 0.4614 |
| ACEMg - Placebo 3 months after ff. | Planned electrode length | -4.1319 | 4.3692 | -12.9433 | 4.6795 | 0.3496 |
|  | Used electrode length | -5.2072 | 4.5336 | -14.3502 | 3.9357 | 0.2571 |
| ACEMg - Placebo 6 months after ff. | Planned electrode length | -4.2882 | 4.4318 | -13.2258 | 4.6494 | 0.3387 |
|  | Used electrode length | -5.5762 | 4.5314 | -14.7147 | 3.5623 | 0.2252 |
| ACEMg - Placebo 9 months after ff. | Planned electrode length | -4.6648 | 4.2773 | -13.2908 | 3.9611 | 0.2815 |
|  | Used electrode length | -6.0982 | 4.3565 | -14.8839 | 2.6875 | 0.1687 |
| ACEMg - Placebo 12 months after ff. | Planned electrode length | -1.1122 | 4.1561 | -9.4938 | 7.2694 | 0.7903 |
|  | Used electrode length | -2.7661 | 4.4194 | -11.6786 | 6.1465 | 0.5347 |


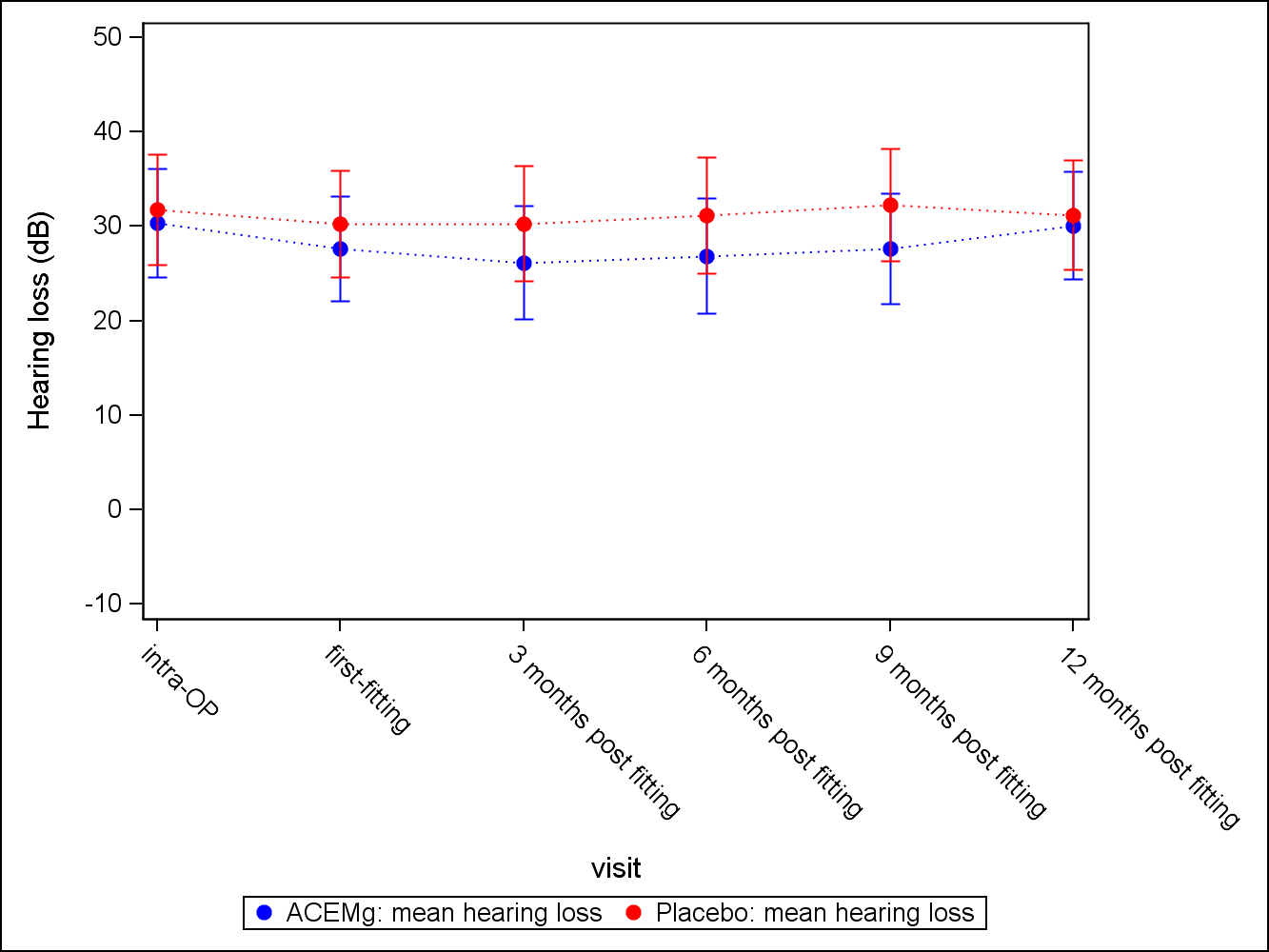


Figure 6: Estimated mean hearing loss at all time points with 95%-confidence intervals based on the mixed model for repeated measures adjusted for the planned electrode length, see table 14.

Supplement 7: The table includes all adverse events and it gives all descriptive analyses regarding safety.

| **CTC grade** | **Treatment** | **Patient** | **Serious** | **SOC** | **PT** | **Outcome** | **Reporter causality to study drug** | **Action taken regarding study medication** | **Duration of AE (in days)** |
| --- | --- | --- | --- | --- | --- | --- | --- | --- | --- |
| 1 | ACEMg | 01-011 | yes | General disorders and administration site conditions | Chest pain | completely recovered/back to baseline conditions | no | no action | 7 |
| 1 | ACEMg | 01-025 | no | Gastrointestinal disorders | Nausea | completely recovered/back to baseline conditions | yes | withdrawn/stopped | 1 |
| 1 | ACEMg | 01-036 | no | Gastrointestinal disorders | Nausea | completely recovered/back to baseline conditions | no | withdrawn/stopped | 6 |
| 1 | ACEMg | 01-036 | no | Gastrointestinal disorders | Vomiting | completely recovered/back to baseline conditions | no | withdrawn/stopped | 5 |
| 1 | ACEMg | 01-036 | no | Ear and labyrinth disorders | Vertigo | completely recovered/back to baseline conditions | no | withdrawn/stopped | 6 |
| 1 | ACEMg | 01-038 | no | Gastrointestinal disorders | Gastric disorder | completely recovered/back to baseline conditions | yes | withdrawn/stopped | 0 |
| 1 | ACEMg | 01-052 | no | Gastrointestinal disorders | Gingival bleeding | completely recovered/back to baseline conditions | yes | withdrawn/stopped | 11 |
| 1 | ACEMg | 01-053 | no | Gastrointestinal disorders | Nausea | completely recovered/back to baseline conditions | yes | withdrawn/stopped | 0 |
| 1 | Placebo | 01-016 | yes | Gastrointestinal disorders | Flatulence | completely recovered/back to baseline conditions | yes | withdrawn/stopped | 69 |
| 1 | Placebo | 01-016 | yes | Gastrointestinal disorders | Nausea | completely recovered/back to baseline conditions | yes | withdrawn/stopped | 8 |
| 1 | Placebo | 01-024 | yes | Investigations | Blood thyroid stimulating hormone increased | completely recovered/back to baseline conditions | yes | withdrawn/stopped | 11 |
| 1 | Placebo | 01-026 | yes | Gastrointestinal disorders | Diarrhoea | completely recovered/back to baseline conditions | yes | withdrawn/stopped | 34 |
| 1 | Placebo |  |  | Skin and subcutaneous tissue disorders | Alopecia | completely recovered/back to baseline conditions | yes | withdrawn/stopped | 54 |
| 1 | Placebo | 01-034 | no | Gastrointestinal disorders | Diarrhoea | completely recovered/back to baseline conditions | yes | withdrawn/stopped | 3 |
| 1 | Placebo | 01-034 | no | Gastrointestinal disorders | Nausea | completely recovered/back to baseline conditions | yes | withdrawn/stopped | 2 |
| 1 | Placebo | 01-034 | no | Gastrointestinal disorders | Constipation | completely recovered/back to baseline conditions | no | withdrawn/stopped | 2 |
| 1 | Placebo | 01-037 | no | Gastrointestinal disorders | Gastrointestinal disorder | completely recovered/back to baseline conditions | no | withdrawn/stopped | 3 |
| 1 | Placebo | 01-039 | no | Gastrointestinal disorders | Diarrhoea | completely recovered/back to baseline conditions | yes | withdrawn/stopped | 6 |
| 1 | Placebo | 01-039 | no | Gastrointestinal disorders | Nausea | completely recovered/back to baseline conditions | yes | withdrawn/stopped | 4 |
| 1 | Placebo | 01-055 | no | Gastrointestinal disorders | Vomiting | completely recovered/back to baseline conditions | yes | unknown | 0 |
| 2 | ACEMg | 01-017 | no | Ear and labyrinth disorders | Ear pain | completely recovered/back to baseline conditions | no | withdrawn/stopped | 10 |
| 1 | ACEMg | 01-017 | yes | Ear and labyrinth disorders | Ear pain | completely recovered/back to baseline conditions | no | no action | 5 |
| 1 | Placebo | 01-026 | yes | Vascular disorders | Hypertension | recovered with sequel | no | withdrawn/stopped | 64 |
| 3 | ACEMg | 01-060 | yes | Neoplasms benign, malignant and unspecified (incl cysts and polyps) | Breast cancer | not recovered (persistently not changing) | no | not applicable | . |
| 1 | Placebo | 01-009 | yes | Injury, poisoning and procedural complications | Upper limb fracture | recovered with sequel | no | withdrawn/stopped | 0 |
| 1 | Placebo | 01-010 | yes | Nervous system disorders | Cerebrospinal fluid leakage | completely recovered/back to baseline conditions | no | withdrawn/stopped | 5 |
| 1 | Placebo | 01-010 | yes | Ear and labyrinth disorders | Tympanic membrane perforation | completely recovered/back to baseline conditions | no | no action | 183 |
| 1 | Placebo | 01-018 | yes | Nervous system disorders | Cerebrovascular accident | unknown | no | not applicable | . |
| 4 | Placebo | 01-048 | yes | Cardiac disorders | Cardiac arrest | completely recovered/back to baseline conditions | no | withdrawn/stopped | 0 |
